# Modality-Specific Perceptual Learning of Vocoded Auditory versus Lipread Speech: Different Effects of Prior Information

**DOI:** 10.3390/brainsci13071008

**Published:** 2023-06-29

**Authors:** Lynne E. Bernstein, Edward T. Auer, Silvio P. Eberhardt

**Affiliations:** Speech, Language, and Hearing Sciences Department, George Washington University, Washington, DC 20052, USA

**Keywords:** speech perception, multisensory, perceptual learning, lipreading, vocoded speech, word learning, spoken language processing, speech perception training

## Abstract

Traditionally, speech perception training paradigms have not adequately taken into account the possibility that there may be modality-specific requirements for perceptual learning with auditory-only (AO) versus visual-only (VO) speech stimuli. The study reported here investigated the hypothesis that there are modality-specific differences in how prior information is used by normal-hearing participants during vocoded versus VO speech training. Two different experiments, one with vocoded AO speech (Experiment 1) and one with VO, lipread, speech (Experiment 2), investigated the effects of giving different types of *prior* information to trainees on each trial during training. The training was for four ~20 min sessions, during which participants learned to label novel visual images using novel spoken words. Participants were assigned to different types of prior information during training: Word Group trainees saw a printed version of each training word (e.g., “tethon”), and Consonant Group trainees saw only its consonants (e.g., “t_th_n”). Additional groups received no prior information (i.e., Experiment 1, AO Group; Experiment 2, VO Group) or a spoken version of the stimulus in a different modality from the training stimuli (Experiment 1, Lipread Group; Experiment 2, Vocoder Group). That is, in each experiment, there was a group that received prior information in the modality of the training stimuli from the other experiment. In both experiments, the Word Groups had difficulty retaining the novel words they attempted to learn during training. However, when the training stimuli were vocoded, the Word Group improved their phoneme identification. When the training stimuli were visual speech, the Consonant Group improved their phoneme identification and their open-set sentence lipreading. The results are considered in light of theoretical accounts of perceptual learning in relationship to perceptual modality.

## 1. Introduction

Speech perception training research has a long history in clinical, educational, and basic science contexts. Training studies have been carried out with speech stimuli across perceptual modalities AO [1], VO [2], and audiovisual (AV) [3], and even haptic [4] and vibrotactile modalities [5], with work dating even back to the 1920s [6]. Effective training has long been seen as having potential to ameliorate the effects of hearing loss in children [2] and adults [7,8] and to improve perception of a second language [9]. Across varied perceptual modalities and goals for training, training tasks have mostly followed traditional approaches. This study was concerned with the possibility that the training task itself might need to be different to obtain perceptual learning across AO speech reduced through vocoding versus VO speech training modalities.

The framework for the traditional training approaches has been described in terms of the contrast between so-called “synthetic” versus “analytic” training paradigms. *Analytic* training mostly refers to phoneme category training with isolated nonsense syllables or words, and *synthetic* training refers to training with connected speech, mostly with isolated sentences or phrases [10,11] but also with connected texts [12]. Analytic training recognizes that languages have relatively small phoneme inventories by which they convey all the words in the language. So, improving phoneme discrimination and identification has been thought to be potentially efficient and effective. However, synthetic training has been seen as more ecologically valid. It is thought to engage the listener in diverse strategies such as using semantic context and guessing [10,11,12]. We have been interested in the possibility that training with visual speech may improve audiovisual speech recognition in noise, particularly for older adults with hearing loss [13,14,15]. However, this study focused on whether there is evidence of modality-specific requirements for training in younger adults with normal hearing.

Recent research on speech perception training has made use of findings from cognitive neuroscience that provide mechanistic theories of speech perceptual learning and suggest diverse approaches to training, e.g., [16,17,18]. The current study followed up on a line of research that investigated how top-down internal feedback might affect perceptual learning of vocoded speech [17]. Vocoded speech has been passed through bandpass filters to obtain the energy in each pass band and then has been reconstituted by multiplying the energy in each band by a noise band or a sinusoid and adding the bands together [19,20]. Although vocoding can result in highly intelligible speech, training studies have been concerned with learning when the speech is degraded. Perceptual learning with vocoded speech has been demonstrated repeatedly [21], where perceptual learning is “long-lasting changes to an organism’s perceptual system that improve its ability to respond to its environment” [22]. In this study, we were concerned with perceptual learning of this kind. Thus, we were not concerned with types of very brief learning such as “recalibration” [23], which persists for about six trials during an experiment. Learning may be quite rapid yet durable with vocoded speech [17,24,25,26,27]. Of particular interest here, the rate of vocoder learning has been shown to increase if knowledge of the vocoded words is provided in advance of hearing them [24,25]. Learning with generalization has been shown with isolated words, novel nonwords, and meaningful or nonsense sentence stimuli [17,24,26,27].

Similar perceptual learning goals exist for lipreading training as for vocoder training, that is, durable learning that generalizes to untrained speech. Normal-hearing adults differ widely in their ability to accurately lipread words in connected speech [28,29,30,31]. On average, they recognize only 10 to 15% of the words in sentences, although some with very good lipreading ability can recognize over 50%. Why these individual differences exist in adults who have experienced normal hearing is not understood. However, the typical adult with age-related acquired hearing loss would likely benefit from being able to lipread more accurately, particularly in noisy social situations in which the talker can be seen as well as heard [13]; therefore, there have been numerous attempts over decades to train lipreading in these adults, with at best modest results [3,13,32]. Demonstration of a successful training method for lipreading could have real-world applications. Here, we sought evidence for whether prior knowledge could be effective with a training paradigm that was applied across vocoded or visual speech with normal-hearing participants.

Mechanisms of vocoded versus visual speech learning. During the prior information training paradigm for vocoded speech, participants are able to first read the words in the spoken vocoded stimulus [24,25]. Learning is more rapid than with no prior information, or when printed information follows the stimulus. The prior information effects are attributed by Davis and colleagues [17,24,26,27] to mechanisms described by predictive coding theory (PCT) [33,34]. According to PCT, perceptual learning entails top-down internal predictions of stimulus information and the updating of those predictions when they do not match bottom-up stimulus representations. The PCT explains why lexical knowledge drives greater and more rapid perceptual learning with better generalization to untrained words than if the information is given after the speech. To investigate the neural substrates for the prior knowledge effect, Davis and Sohoglu carried out a neurophysiological study, in which prior lexical knowledge and stimulus clarity (number of noise bands) of vocoded speech were manipulated. Their results suggest that both temporally prior lexical knowledge and stimulus clarity modulate activity in the temporal gyrus proximal to Heschl’s gyrus (Figure 5a, [27]). That is, their explanation was that printed text prior to vocoded speech generates a lexical candidate prediction (top-down feedback) for upcoming vocoded speech stimuli and that the predictions are confirmed or disconfirmed by bottom-up auditory sensory representations, with subsequent updating of the predictions [17,27,35].

However, the mechanistic account of vocoder learning cannot be applied directly to visual speech stimuli, which are processed through visual pathways to high-level visual representations. Indeed, we have shown in an imaging study that visual speech is processed by higher-level visual representations [36]. We contrasted video and animated point light stimuli of a talking versus a non-speech gesturing face versus scrambled stimuli, and we investigated activation in candidate regions of interest (ROIs) identified with localizers for the fusiform face area (FFA), the lateral occipital complex (LOC), and the visual motion area (MT). Distinct left cortex activation of the posterior superior temporal sulcus (pSTS) and its adjacent middle temporal gyrus (MTG) cortex was obtained for visual speech uniquely, and we dubbed the area the “temporal visual speech area” (TVSA). We then carried out a visual speech, EEG mismatch negativity study and demonstrated change detection consistent with phonemic representations in the location of the left TVSA [37]. A subsequent study obtained evidence for lexical representations of visual speech in TVSA [38]. Thus, the PCT explanation by Davis and colleagues [17] for the efficacy of prior printed information through targeting auditory lexical representations does not seem compatible with the functional neuroanatomy of visual speech processing [39,40].

PCT is not the only relevant theoretical account of perceptual learning. The reverse hierarchy theory (RHT) [41,42] posits that what is learned is dependent on the flow of information through feedback and feedforward cortical pathways. Like PCT, RHT considers the hierarchical organization of sensory-perceptual systems of the cerebral cortex to comprise bottom-up representations of increasing generality and non-invariance at higher cortical levels [43,44,45,46]. However, RHT offers an alternative mechanism for perceptual learning across both auditory and visual speech.

RHT [42,47,48] posits that whenever possible, perceptual tasks rely on the highest available neural representations (i.e., the highest-level generalizations or categories). When a perceptual task cannot be carried out based on the highest or most general available representations, external feedback may be needed to guide attention to information in stimulus representations at lower levels of the cortical hierarchy, be they auditory or visual. That is, the perceiver may implicitly or explicitly carry out a reverse search to access lower-level perceptual representations such as prelexical phonetic or phonemic representations. RHT posits that perceptual learning entails remapping lower-level representations such as phonetic details or features that are needed by a task to higher-level categories, representations, or generalizations. A reverse search is a type of internal feedback. Thus, RHT suggests that external feedback may be needed for perceptual learning, but a reverse search is carried out within the bottom-up modality-specific stimulus processing pathway.

The RHT and PCT differ in their implications for semantic processing during perceptual learning. PCT for vocoder learning suggests that feedback is initiated to lower-level stimulus representations by lexical knowledge, while RHT suggests that semantic processing interferes with perceptual learning because perceptual learning requires relinquishing attention to semantic representations [48]. According to Ahissar (2009), “we cannot have accurate within-category discrimination, with concomitant semantic processing” (p. 288). That is, if the perceptual learning task requires learning and access to representations that can differentiate within existing perceptual categories (e.g., within ambiguous vocoded speech or viseme-like categories), then a semantic task such as recognizing printed words or learning new words will interfere with perceptual learning.

In particular, consideration of RHT and PCT suggests that there may be differences in the requirements for perceptual learning with visual versus vocoded speech: the type of information available to the trainee may significantly affect learning. Nevertheless, some type of external feedback or information is likely to be needed when stimuli are not obvious [49], particularly if adults are to learn difficult stimuli such as visual speech [50,51,52,53]. In this study, we manipulated the type of prior information during training with novel words to determine whether prior information type affects performance during training and what is learned.

This study. We investigated different types of prior knowledge during training with vocoded (Experiment 1) or visual (Experiment 2) speech. However, because existing lexical knowledge appears to automatically induce internal feedback through auditory speech processing pathways, and because existing semantic knowledge may interfere with perceptual learning, our training task used novel nonsense words. Participants were given the task of learning to label novel visual objects with novel words (e.g., “tethon”) [54,55,56]. Prior knowledge of novel vocoded words has been shown to be effective in vocoder training [25]. Here, regardless of modality, the use of novel words could defend against reliance on existing lexical and semantic representations and thereby encourage attention to the perceptual stimuli during the label-learning task.

**Figure 1:**
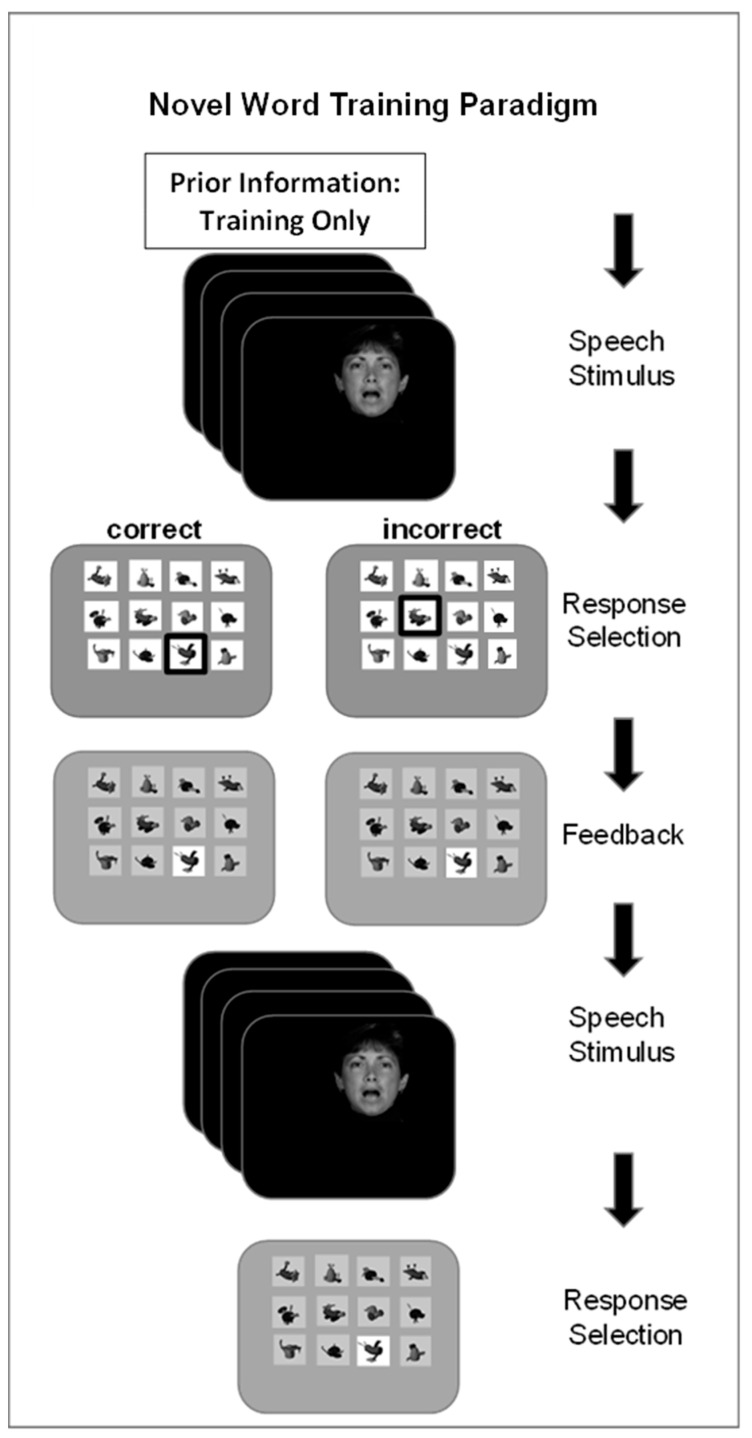
Outline of the Novel Word Training and Testing. When prior information was presented, it was presented first on each trial, followed by the speech stimulus. Then, a matrix of 12 nonsense pictures was displayed, and the participant clicked on a picture to label the word. If the selection was correct, it was highlighted; if it was incorrect, the correct picture was highlighted. The spoken stimulus was then repeated, and the participant selected the correct picture to move on to the next trial. The test that followed each training set comprised only the stimulus followed by the matrix of pictures and the participant’s response. There was no prior information, and the trial terminated with the participant’s response, without any feedback. Additionally, half of the training words were replaced by foils.

We previously applied the paradigm here to show that vocoded and visual versions of novel words can be learned to a high level of accuracy without prior information [54,56]. However, generalization to phoneme identification was modest. Here, we used three different types of prior information during training by different groups of trainees in an attempt to improve generalization. Figure 1 illustrates the novel word paradigm. When prior information was presented, it was presented first on each trial, followed by the speech stimulus. Then, a matrix of 12 nonsense pictures was displayed, and the participant clicked on a picture to label the word. If the selection was correct, it was highlighted; if it was incorrect, the correct picture was highlighted. The stimulus was then repeated, and the participant selected the correct response to move on to the next trial. The test that followed each training set omitted prior information, presented the stimulus only once, and terminated the trial after the response, with no feedback given.

A group referred to as the “Word Group” received a prior printed version (e.g., “tethon”) for each of the 12 to-be-learned novel words. We expected that the prior lexical information would be effective for vocoded speech because of the tight coupling of lexical processing to auditory representations in normal-hearing adults. However, because normal-hearing adults rely on auditory spoken language, we reasoned that lexical processing through reading printed information would interfere with the learning of visual speech. Our interpretation of RHT suggested that training with visual speech would be more effective with knowledge of the phonemes in the visual stimuli but without lexical knowledge. Full lexical knowledge could initiate reliance on auditory processing mechanisms. To reduce lexical information but present phonemic information, Consonant Group trainees received prior knowledge in the form of only the consonants in each training word (e.g., “t_th_n”).

We also compared word or consonant prior information with results from having no prior information (Experiment 1, auditory-only, AO, Group; Experiment 2, visual-only, VO, Group) and across groups that were trained with reduced cross-modal information (Lipread Group, Experiment 1; Vocoder Group, Experiment 2). That is, in each experiment, there was a group that received prior information in the modality of the training stimuli from the other experiment. The cross-modal groups were a partial control for the Consonant Group’s information reduction relative to prior word information. Generalization was tested in Experiment 1 on vocoded speech using forced-choice phoneme identification, and generalization was tested in Experiment 2 on visual speech using forced-choice phoneme identification and open-set lipreading. We did not carry out pre- and posttesting with vocoded sentence stimuli because listening to real vocoded sentences before training could induce significant learning [25], which could interfere with observing group differences during and after training.

## 2. Experiment 1: Vocoded Speech

We investigated the effects of prior knowledge on novel word training and testing with vocoded speech and the generalization of training to forced-choice phoneme identification across pre- and posttraining tests. Participants were assigned to Word, Consonant, AO, or Lipread Groups. The Lipread Group first saw the visual speech and then heard the same speech vocoded during novel word training.

### 2.1. Materials and Methods

**Participants**. Participants were screened to have normal pure-tone auditory thresholds. They were randomly assigned to training groups: Consonant Group, ages 18–26 yrs, mean 21.0 yrs, 2 males, N = 17; Word Group, ages 18–29 yrs, mean 21.0 yrs, 5 male, 1 dropped out during training (16–11), N = 14; and Lipread Group, ages 20–28 yrs, mean 22.0 yrs, 1 male, N = 16. The AO Group was ages 20–39 yrs, mean 25.7 yrs, 2 males, 2 dropped out, N = 18. The Control group was ages 20–29 yrs, mean 22.4 yrs, N = 10. All of the participants provided written informed consent as approved by the George Washington Institutional Review Board, except for the no-training Control Group and the Auditory-Only (AO) Group. The latter were tested at the House Research Institute, Los Angeles (and consented there with approval of its Institutional Review Board). All of their results were reported previously [54].

**CVCVC novel training word stimuli.** All of the novel word stimuli that were used in training were described in detail previously [54,55]. An abbreviated description follows. CVCVC (C = consonant, V = vowel) novel words were spoken by one female talker. The stimuli were modeled on English phonotactics (i.e., the sequential phoneme speech patterns in English) using Monte Carlo methods [54]. The stimuli had varied vowels, and across stimuli, the 24 English consonants were used. The words were modeled to be mutually visually distinct (via lipreading) and visually unique from real English words (i.e., the words were designed to not be mistaken as real words if they were lipread without accompanying audio). For example, the novel word *mucker* was not included in the set because the visual stimulus could be mistaken for the real word *pucker*, inasmuch as the phonemes /p, m/ are visually similar [57]. Previous studies showed that the stimulus development method was successful in creating mutually distinct, highly learnable novel words [54,56].

The novel words were allocated to four lists of 12 words for training and to four lists of 6 words that were used as foils during testing. No words were repeated across lists. Additionally, two 49-item lists of words that were not used in training were used for pre- and posttraining forced-choice consonant identification. Different six-item lists were used for practice before each of the consonant identification tests.

During training by the Word Group, the novel word spellings were modeled on possible English spellings, for example, “cherfing” and “jobit” (see Appendix A). The Consonant Group saw each stimulus printed with consonants in the format “C_C_C,” or with CC for consonant clusters. Consonants were presented as orthography, not phonemic transcriptions; for example, “th” and “sh” were used.

**Figure 2:**
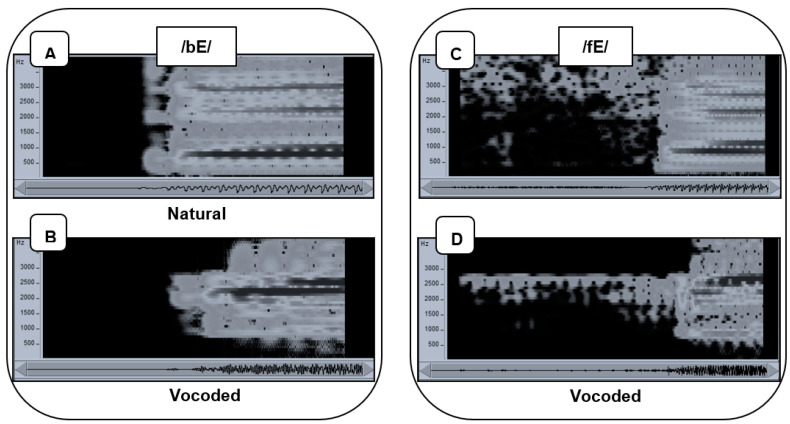
Examples of natural and vocoded speech. Beneath each natural stimulus (**A**,**C**) is its vocoded version (**B**,**D**, respectively).

**Vocoded speech stimuli.** The vocoded acoustic speech was the output of the F2 vocoder from our previous studies [54,56,58] (Figure 2). The vocoder was a real-time device that had thirteen channels focused around the second speech formant, with 120-Hz bandpass filter frequencies spaced 150 Hz apart from 825 to 2625 Hz. Two additional filters passed high frequencies. One was a bandpass filter centered at 3115 Hz with a 350 Hz bandwidth, and the other was a highpass filter with 3565 Hz cutoff. The energy passed by each of the filters modulated fixed-frequency sinewaves that were summed prior to presentation as the acoustic stimulus. This stimulus retained the gross spectral-temporal amplitude information of the original acoustic stimulus and eliminated finer details such as the fundamental frequency and the natural spectral tilt of the vocal tract. The vocoder in this experiment was shown in a forced-choice phoneme identification experiment to produce 56.6% correct consonants (using all initial English consonants) and 81.3% correct vowels (using 15 different vowels) [58]. In the same experiment, lipreading performance with the visual recordings of the same stimuli was 47.8% correct consonants and 51.3% correct vowels. The vocoder consonant identification was deemed reasonably reduced for this study of learning across modalities.

**Novel picture stimuli**. The novel objects in the training paradigm were from the fribbles image set (https://sites.google.com/andrew.cmu.edu/tarrlab/stimuli, accessed with new address on 24 June 2023) [59] (Stimulus images courtesy of Michael J. Tarr, Carnegie Mellon University, http://www.tarrlab.org/ accessed on 27 March 2023) and had been used previously for the novel word training paradigm [54,55,56]. Fribbles comprise 12 species with distinct body “core” shape and color, with 81 exemplars per species obtained by varying the forms of each of four appendage parts. From the available images, four lists of 12 images each were created such that each list used three different body forms and no duplicated appendage forms, resulting in highly distinct images within each set. No appendage was repeated across lists. Our previous studies showed that the novel pictures could be learned to high accuracy levels in the novel word training paradigm.

**Novel word training and testing procedure.** There were four separate novel word training days. On each training day, the task was to learn 12 unique novel words for labeling 12 unique novel pictures. Figure 1 outlines the events during a training trial. If prior information was given, it was presented before the spoken stimulus. Different participant groups received different prior information as either the printed novel word (Word Group) (e.g., “tethon”), only the consonants in the printed word (Consonant Group) (e.g., “t_th_n”), the visual speech corresponding to the vocoded token (Lipread Group), or no prior information (AO Group). Groups with prior information received their information on the first presentation of the stimulus on every training trial.

On each training trial, after the prior information and the stimulus were presented, a matrix of 12 novel pictures appeared on the computer monitor. The participant selected a picture with a mouse click, and all but the correct selection were darkened on the screen. Then, the spoken stimulus was repeated, the novel picture matrix was presented with the incorrect choices darkened, and the participant clicked on the correct choice to continue to the next trial. A training block comprised two repetitions of 12 words per list presented in pseudorandom order. There were three blocks per list, that is, six training trials per word. Practice carrying out the task was given for the first training list using six stimuli that were only for practice. The pictures’ positions in the matrix were re-randomized for each training and test trial.

Testing followed training after a few-minute pause. The trials comprised presentation of the vocoded novel words without any prior information, followed by presentation of the matrix of novel pictures. Selection of a novel picture triggered the next trial. Six of the trained words and all 12 of the trained images were used. There were six untrained foil CVCVC novel words that were paired with the novel images of the discarded trained words. During the test block, there were four presentations of the twelve (6 old and 6 new) stimuli in pseudorandom order.

**Pre- and posttraining CVCVC consonant identification stimuli and procedure**. Before and after training, participants carried out forced-choice consonant identification. The stimuli were CVCVCs (C = consonant, V = vowel) that were not used during training. For each stimulus, the task was to identify its three consonants using a forced-choice response. The ARPABET [60] system was used to label the consonants /b, d, f, g, h, k, l, m, n, p, r, s, t, v, w, y, z, C, D, G, J, S, T, Z/ (which correspond to the International Phonetic Alphabet, /b, d, f, g, h, k, l, m, n, p, r, s, t, v, w, j, z, tʃ, ð, ƞ, dƷ, ʃ, Ɵ, Ʒ/). The computer keyboard was used for single key-press forced-choice responding. During practice trials, an incorrect key-press elicited a message that the selection was wrong and prompted the participant to try again. During testing, the computer monitor displayed the ARPABET symbols and corresponding word examples near the top of the screen. The three CVCVC consonant positions were marked on the computer screen’s center (in large font with “__-__-__”), and the participants used the keyboard to fill in the blanks. They could backspace and correct mistakes. No feedback was given. Stimulus lists had 47 or 48 CVCVCs and were counter-balanced across participants and testing sessions.

**Procedure summary.** Participants sat in a sound-treated room. Stimuli were presented using a computer, monitor, and a calibrated high-performance loudspeaker.

**Figure 3:**
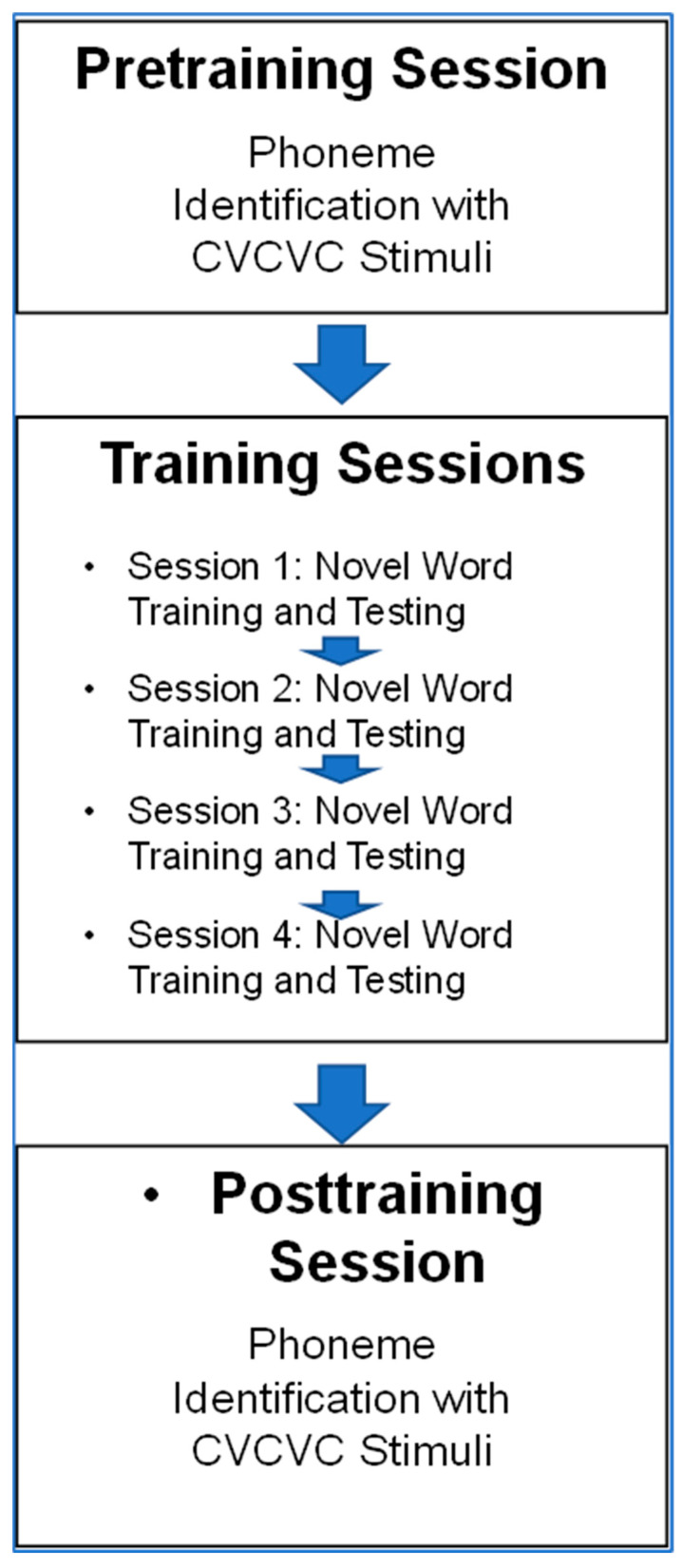
Outline of Experiment 1 procedures. Pre- and posttraining sessions and training sessions were carried out on different days.

Figure 3 illustrates the task order across sessions. Before training, participants were tested on forced-choice consonant identification with a CVCVC novel word list not used in training. The participants returned on four separate days to train and test with a different novel 12-word list per day. Training lasted for generally less than 25 min per day, and testing per day was for about 6 min. Then, on a different day, participants were posttested on consonant identification with a different set of CVCVC novel words than words they heard before. The Control Group received only forced-choice consonant testing on two separate days at least one week apart.

**Data.** The novel word training data were the individual correct/incorrect (0/1) scores from training and testing parts of the paradigm, excluding foil trials. The pre- and posttraining consonant identification data were the individual correct/incorrect (0/1) scores per consonant and position (i.e., initial, medial, and final).

**Analyses.** Generalized linear mixed models (GLMMs) [61] were applied. The GLMMs were computed as logistic regression with the binomial distribution, resulting in log odds regression coefficients. The exponentiated log odds minus 1 multiplied by 100 are the percent odds of an increase or decrease for a one-unit change in a predictor. *Wald’s z* and *p-values* are reported in addition to the model coefficients. The data and R code are available at https://osf.io/v5z8x/ accessed on 25 June 2023. The lme4 package [62] and the R language for statistical computing [63] within RStudio [64] were used. The effects package [65] was used in generating effects plot graphics. Effects plots show the fixed (i.e., exact) factors.

### 2.2. Experiment 1: Vocoded Speech Results

The novel word training and retention results are presented first, followed by the test–retest phoneme identification results.

**Figure 4:**
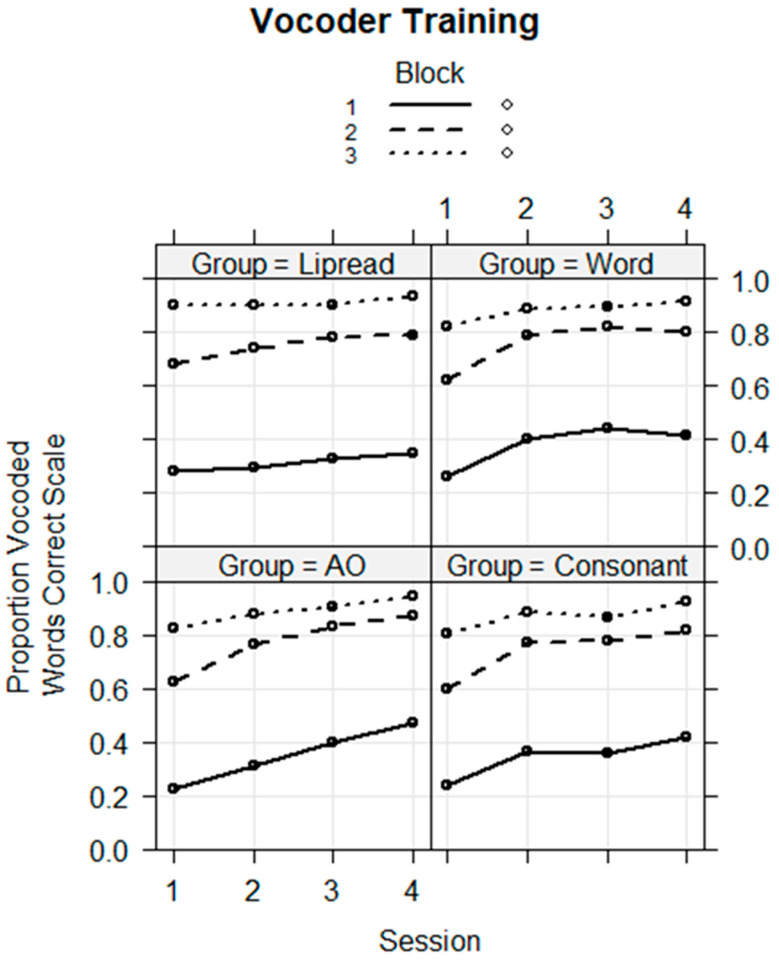
Effects plot: Modeled vocoder training. The fixed effects are shown for the individual groups, sessions, and blocks. The scale is proportion of correct words.

**Novel word training.** The training data were analyzed to determine whether the groups were on average different, and whether performance changed across sessions. A mixed-effects logistic regression model was fitted with the fixed factors of group (Word, Consonant, Lipread, and AO), session (dummy-coded four sessions), training block (dummy-coded three training blocks), and their higher-way interactions. The random factors were the stimulus and the subject intercepts. The AO Group, Session 1, and Block 1 were the references in the model. The dependent variable was the individual trial correct or incorrect scores (1/0). The model with all higher-way interactions did not converge. A model that included only all of the two-way interactions converged and is shown in Table 1. Figure 4 shows the model’s fixed-effects plot for performance during training.

The coefficients in Table 1 show that on average, none of the groups differed from the AO reference group, that the second, third, and fourth sessions were all significantly more accurate on average than Session 1, and that Blocks 2 and 3 were significantly more accurate on average than Block 1. However, there were significant interaction effects. There were significant interactions of Sessions 3 and 4 with Block 2 (Session 3 × Block 2, *coef* = 0.268, *z* = 2.278, *p* < 0.05; Session 4 × Block 2, *coef* = 0.252, *z* = 2.122, *p* < 0.05), suggesting that in these sessions, learning was faster during Block 2. However, we did not regard these effects as particularly important, because group was not a factor in the interactions.

With regard to the group by session interactions, the Lipread Group, which trained with the cross-modal lipreading stimulus prior to the vocoded speech, performed more poorly than the AO Group (Session 2, *coef* = −0.385, *z* = −2.707, *p* < 0.01; Session 3, *coef* = −0.614, *z* = −3.744, *p* < 0.001; and Session 4, *coef* = −0.821 *z* = −4.891, *p* < 0.001), and the Word Group performed more poorly than the AO group in Session 4 (*coef* = −0.455, *z* = −2.685, *p* < 0.01). These results suggest that the Lipread Group was paying attention to their prior information but having difficulty using it relative to the AO Group, which received no prior information. The Word Group interaction suggests that they may have been paying less attention in Session 4 during training.

**Figure 5:**
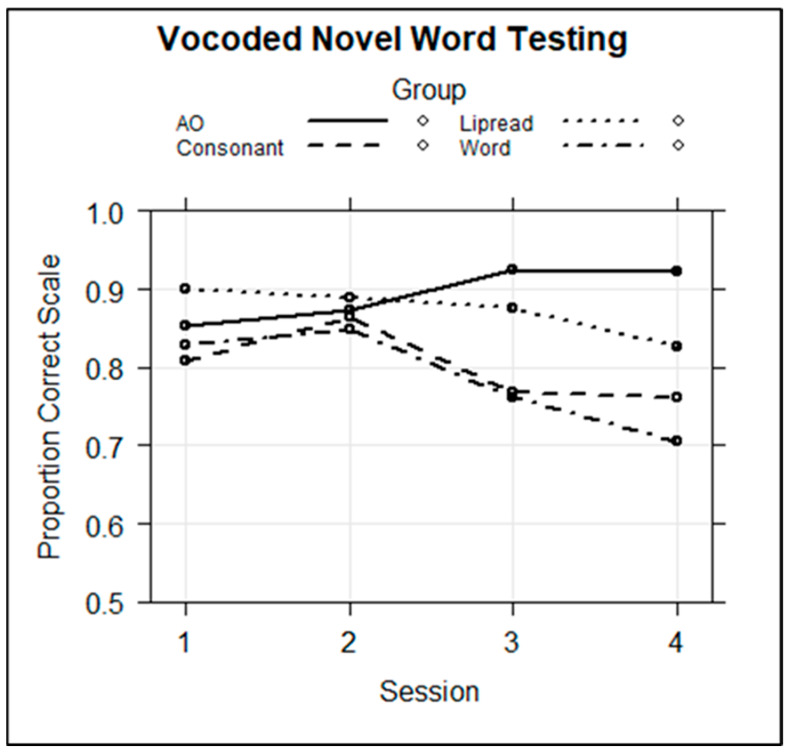
Fixed-Effects Plots: Modeled Retention Results for Vocoded Novel Word Labeling in Experiment 1.

**Novel word retention test.** Following training and within the same session, participants were tested on their retention of novel visual object labeling. A mixed-effects logistic regression model was computed with the fixed factors of group, session, and their interaction and with the individual trial correct or incorrect (1/0) scores as the dependent variable. The random effects were the subject and stimulus intercepts. The AO Group and Session 1 were the references. Table 2 displays the full regression model. Figure 5 shows the fixed-effects results across groups and sessions.

The results revealed that there were no significant effects for group, session, or group by Session 2. However, there were significant negative regression coefficients for the group by session interactions for the Word (Session 3, *coef* = −1.172, *z* = −2.597, *p* < 0.01; Session 4 *coef* = −1.526, *z* = −3.368, *p* < 0.001), Consonant (Session 3, *coef* = −0.997, *z* = −2.229, *p* < 0.05; Session 4, *coef* = −1.0851, *z* = −2.418, *p* < 0.05), and Lipread (Session 3, *coef* = −0.966, *z* = −2.072, *p* < 0.05; Session 4, *coef* = −1.465, *z* = −3.166, *p* < 0.01) Groups relative to the AO reference group. That is, the groups with prior information all performed worse on the test in Sessions 3 and 4 than did the AO Group. Because Figure 5 suggests that the worst retention was in Session 4 by the Word Group, the model was re-run with the Word Group as the reference. Indeed, this showed that with the Word Group as the reference, the AO Group was unique among the other groups in being more successful at retaining the novel words in Session 4.

### 2.3. Pre- and Posttest Forced-Choice Phoneme Identification

Before and after training, participants performed forced-choice phoneme identification with CVCVC stimuli that were not used in training and were not repeated across the tests. However, before carrying out an analysis of the results, the participants’ performance during training was examined for participants who may not have been engaged in or been able to perform adequately. We reasoned that during training, the participants should have been able to learn at least 8 of the 12 novel words. We did not use the novel word test results for determining performance, because the analyses above had shown that the prior information affected the groups differently at test. When we examined the individuals’ training data, we saw that by the third block of training, some participants were still identifying fewer words than 8. In order to evaluate how prior information affects comparable pre- to posttraining performance, we removed participants if their training accuracy during the third block was less than 8 words for at least two separate training sessions. There were two in each training group who were removed for failing to perform to criterion during training.

To determine whether there was learning with generalization, a mixed-effects logistic regression model was computed with the fixed factors of group, test (pre- vs. posttest), and CVCVC consonant position (initial, medial, final) and all of their interactions. The references were the Control Group, the initial consonant position, and the pretest. The dependent variable was the correct or incorrect (1/0) score for each of the consonant positions for each stimulus per trial. The participant intercepts and the stimulus intercepts and their correlated position slopes were the random factors.

The model’s regression coefficients (Table 3) showed significant group effects such that each of the three groups with prior information performed on average less accurately than did the Control Group (*p* < 0.001). Scores improved across sessions (*coef* = 0.492, *z* = 2.919, *p* < 0.01), and Position 2 (medial) consonants were identified significantly more accurately than Position 1 (*coef* = 0.962, *z* = 3.621, *p* < 0.001).

**Figure 6:**
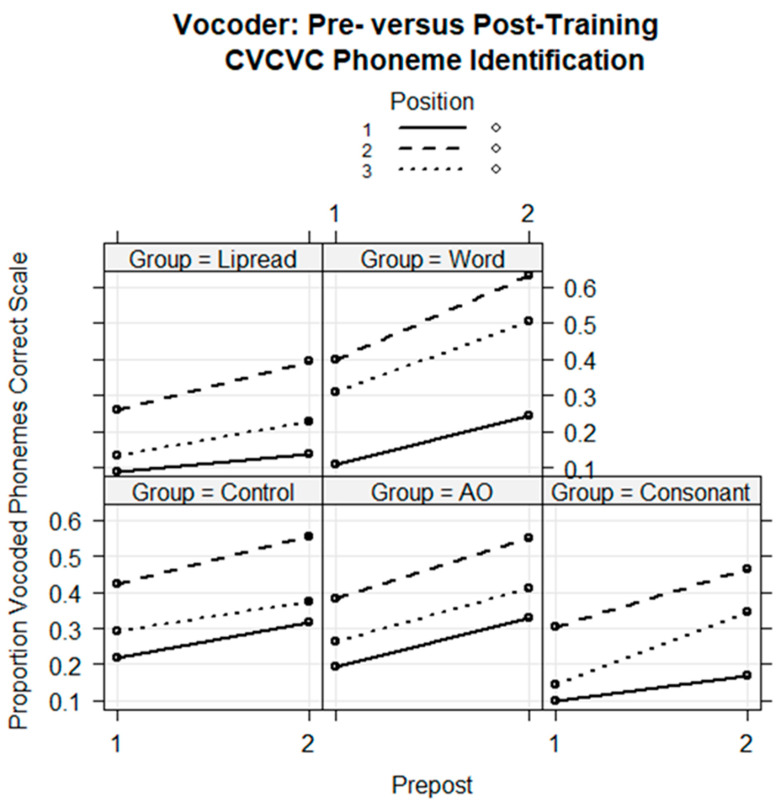
Fixed-Effects Plot: Modeled pre- and posttraining forced-choice consonant identification. The effects are shown for the individual training groups and the Control Group across Pre- and Posttraining sessions and for the different consonant positions.

Of primary interest, there were several significant effects among interactions that involved group. The Word Group benefitted significantly from training relative to the test–retest difference in the Control Group’s scores (*coef* = 0.492, *z* = 2.072, *p* < 0.05). That is, the difference between test and retest for the Word Group was significantly larger than for the Control. However, there was also an interaction of Word Group and medial consonant position (*coef* = 0.918, z = 3.882, p < 0.001) and an interaction of Word Group by final consonant position (*coef* = 0.674, *z* = 2.213, *p* < 0.05), meaning that the Word Group’s increase in medial and final consonant identification after training was larger than were the increases by the Control Group (Figure 6). There were no significant interactions with the AO or Lipread Groups, thus, no indication that their training generalized to consonant identification. However, there was one significant three-way interaction of Consonant Group by session by final consonant position (*coef* = 0.673, *z* = 2.213, *p* < 0.05), showing that the Consonant Group was able to improve its scores on one of the positions.

Examination of the regression model’s marginal means showed that the Word Group’s estimated improvement was, for initial consonants, 13.7 percentage points (p.pt.); medial consonants, 23.3 p.pt.; and final consonants, 19.8 p.pt. The Consonant Group’s difference scores were, for initial, 6.8 p.pt.; medial, 16 p.pt.; and final, 19.9 p.pt. The Control Group’s difference scores were, for initial, 9.6 p.pt.; medial, 12.8 p.pt.; and final, 7.9 p.pt. Word Group means show that the training effects were substantial across CVCVC stimulus positions. The Consonant Group means show that the training had a more limited effect for this group.The Control Group’s substantial test–retest improvement confirmed the need for this group as a comparison to trained groups. 

**Discussion**. In Experiment 1, participants trained to use novel vocoded words for labeling novel pictures. On four training days, they learned a different set of 12 novel words, and they were tested on their retention of the labeling on the same day. The participants knew that they would be tested each day and that there would be new and old novel words during testing. However, by presenting printed words first, then the to-be-learned stimulus words, the Word Group could adopt a strategy of instead learning the relationships between the printed words and the novel pictures, rather than the relationships between the spoken words and the novel pictures. If they did that, their training performance could be highly accurate, but their retention might not be.

Indeed, across training sessions, the Word Group was highly accurate during the training blocks, but they were poorer at novel word retention, significantly so during Sessions 3 and 4. Nevertheless, the Word Group achieved large changes in their phoneme identification, a test of perceptual learning and generalization. These results suggest that lexical knowledge benefitted vocoder learning, consistent with the PCT explanation of vocoder learning [17], even though lexical knowledge interfered with the semantic task of learning the novel words as labels in Sessions 3 and 4. This result seems consistent with the suggestion that perceptual learning requires relinquishing semantic processing, as suggested by the RHT [48]. While the use of novel words likely reduced reliance on existing semantic knowledge, the novel word training task can itself be regarded as a semantic task.

The results across pre- and posttests and training revealed apparent dissociations between performance during training versus performance at test. For example, the Word Group retained fewer novel words during the novel word retention tests but nevertheless demonstrated the greatest perceptual learning. In contrast, the AO group retained the novel words but showed no generalization of learning. However, these types of dissociation between performance during training and learning have been shown to be common. A training task may be constructed so that performance during training can be achieved without any perceptual learning, and on the other hand, no learning may be observed during training, yet perceptual learning can be obtained (for a review, see [66]). Indeed, the learning by the Word Group can be regarded as a type of “task-irrelevant learning” [67] because phoneme learning was *not* required to successfully learn novel words as labels for novel pictures, yet the Word Group significantly improved their phoneme identification. That phoneme learning was *not* required by the task was demonstrated by the AO Group, which was highly successful in its learning to label the novel pictures but showed no training effects on phoneme identification. There was sufficient information in the vocoded novel words for the AO Group to learn to use them to label the novel pictures [54], but there was no evidence that they improved their phoneme identification.

The Consonant Group improved its phoneme identification for final consonants only. The Consonant Group’s learning can be explained by RHT: because learning was selective for final consonants, it may have resulted from a reverse search for the final consonant stimulus information. The Lipread Group, which received cross-modality prior information did not improve its phoneme identification, even though evidence from training results suggests that they paid attention to the prior visual speech. The Lipread Group was considered to be a partial control for the prior information reduction experienced by the Consonant Group, which saw only the printed consonants. The Lipread Group was considered a *partial* control because lipread information did not match the removal of vowel information that the Consonant Group experienced. Nevertheless, the cross-modal prior lipreading information was a reduced stimulus. The results suggest that a reduced stimulus can be helpful but only when the remaining information is unambiguous.

This experiment included a group of untrained controls. Untrained controls are very important in training studies because of the expectation that there will be improvements in performance across pre- and posttraining test sessions, even without training. The use of the Control Group as the reference assured that significant learning by trained groups could be attributed to the learning that occurred through training and not merely to repeated experience in the phoneme identification task.

Overall, Experiment 1 supports the conclusion that the type of prior information provided during training differentially affects perceptual learning and that lexical information is most effective for vocoded speech, consistent with PCT. However, the reduced novel word retention by the Word Group was also consistent with the RHT suggestion that there is a conflict when a semantic task and perceptual learning are both goals.

## 3. Experiment 2: Lipread Speech Materials and Methods

Experiment 1 showed that vocoder learning is sensitive to the type of prior information given during novel word training. Experiment 2 was carried out to determine how the type of prior information affects the learning of visual speech. We expected different effects from those for vocoded speech: the neural evidence that internal lexical feedback targets auditory representations [27] implies that prior lexical knowledge would not necessarily be useful for visual speech stimuli, which are processed through the visual system. Neuroimaging research on visual speech suggests that visual speech may be represented *qua* speech to at least prelexical visual representations [36,39,40,68,69] and even possibly to the level of lexical forms [38,69,70]. Therefore, prior lexical information that may be highly effective for the perceptual learning of vocoded speech may not be effective or may even be an impediment to learning visual speech. Additionally, because adults with normal hearing are generally not expert lipreaders, we expected that their prelexical perception should be the target of learning.

We predicted for Experiment 2 that prior consonant information would promote the most learning with generalization. The consonant information could support prelexical perceptual learning through the mechanisms described by RHT. Additionally, prior consonant information would defend (at least early on in training, before lexical representations were learned) against top-down feedback to auditory lexical representations. Although the experiments in this study were behavioral, contrasts in the pattern of results across vocoder (Experiment 1) versus visual speech (Experiment 2) training could align with the predictions based on the neural studies and thereby provide support for future neural studies of modality-specific perceptual learning.

### 3.1. Materials and Methods

Participants. Participants were screened with a calibrated audiometer to have normal pure-tone auditory thresholds. They were screened with a Snellen chart to have normal visual acuity. They were tested on their lipreading ability [30] and were assigned randomly to training groups according to lipreading score quantiles in an attempt to obtain a range of lipreading abilities in each group. The participant groups were Consonant Group, ages 18−26 yrs, mean 20.7 yrs, 6 males, N = 25; Word Group, ages 18–26 yrs, mean 20.8 yrs, 5 males, N = 21; and Vocoder Group, ages 18–30 yrs, mean 21.6 yrs, 6 males, and three participants subsequently dropped out, leaving N = 20. Data were imported from Eberhardt et al. [56] for the visual-only (VO) Group, ages 18–31 yrs, mean 21.9 yrs, 2 males, and one dropped out, leaving N = 20. There was also a no-training Control Group, ages 20–29 yrs, mean 22.4 yrs, 3 males, and one dropped out, leaving N = 19. All of the participants signed a written informed consent form that was approved by the George Washington University Institutional Review Board. They were paid for their participation.

CVCVC novel training word and phoneme identification stimuli. The novel word training and test stimulus lists and the pre- and posttraining consonant identification lists were the same as in Experiment 1, except that the training words and CVCVC pre- and posttest stimuli were presented as visual speech.

**Figure 7:**
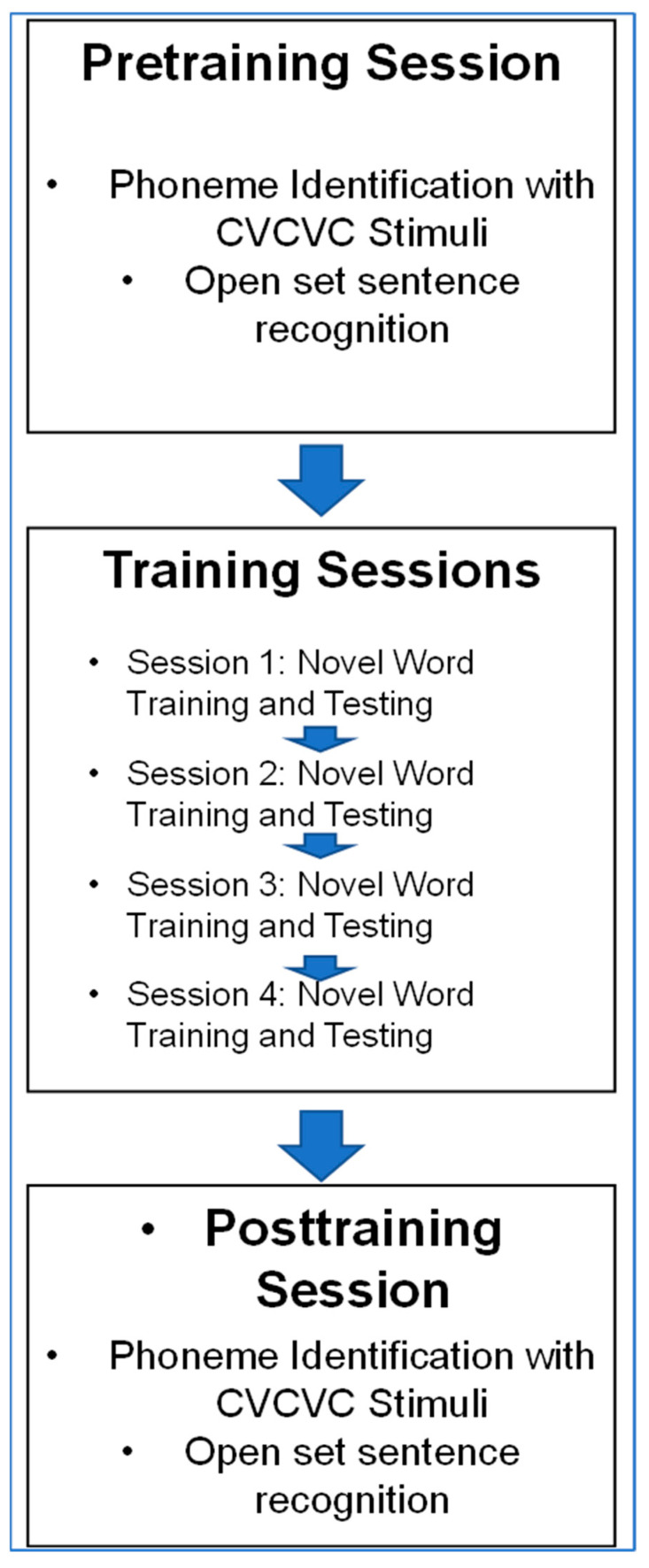
Outline of Experiment 2 procedures. Pre- and posttraining and novel word training were carried out in different sessions. Phoneme identification and open-set sentence recognition were tested in the same sessions.

Novel word training and testing procedure. Figure 7 is an outline of the procedures in Experiment 2. The novel word training and testing procedures were the same as in Experiment 1. The information that was presented prior to novel words during training by the Word and Consonant Groups was the same as in Experiment 1. The cross-modality Vocoder Group received each training word in its vocoded version first and then received its visual speech version. The vocoder was the same as the one in Experiment 1. The VO Group received no additional information.

Pre- and posttest sentences and procedure. In addition to forced-choice phoneme identification with CVCVC stimuli, pre- and posttraining tests included sets of visual-only sentences that were presented for open-set lipreading. Two different 35-sentence lists comprised video recordings of the low-context IEEE sentences [71] spoken by a different talker (male) than the one seen during training. List order was counter-balanced across pre- and posttraining tests and participants. Participants received each sentence once without feedback. Their task was to type whatever they thought the talker had said following each of the stimuli.

Summary of the procedure. The procedures in Experiment 2 matched those in Experiment 1 except for the stimulus modality, the vocoded speech for the cross-modality training group (i.e., Vocoder Group), and the inclusion of sentence stimuli during pre- and posttraining tests.

Data and analyses. The types of data and the analyses were the same as in Experiment 1, except for the open-set sentence data. Those responses were automatically scored for words correct, and the data were analyzed using the proportion of correct words per sentence, with the number of stimulus words as weights in logistic regression analyses.

### 3.2. Experiment 2: Lipread Speech Results

The novel word training and testing results are presented first, followed in order by the pre- and posttraining tests of phoneme identification and lipreading.

**Figure 8:**
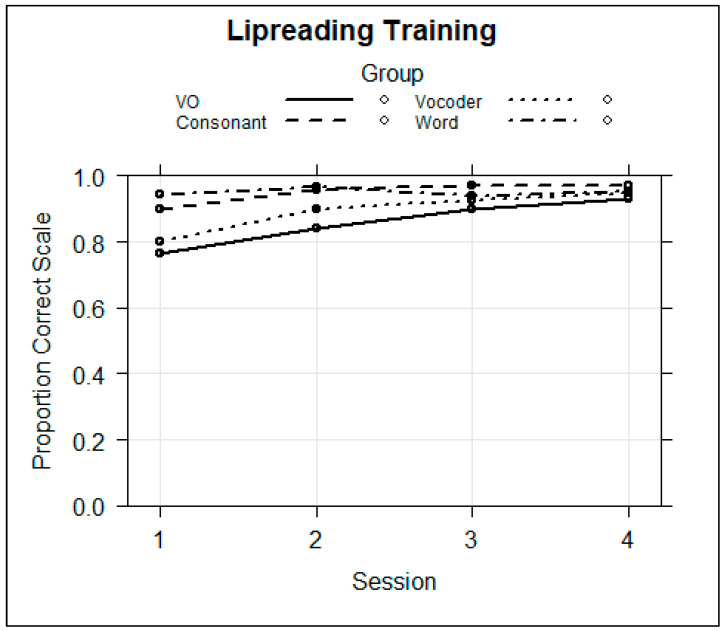
Fixed-Effects plot: Modeled Novel Word Lipreading Training. The effects for lipreading training trials in Block 3 are shown for each of the training groups.

Novel word lipreading training. The training data were analyzed to determine whether the groups were on average different and whether performance changed across sessions. A logistic regression model was fitted with the fixed factors of group, block, and session and all of their interactions and with the random factors of subject and stimulus intercepts. The dependent variable was the individual trial correct or incorrect (1/0) scores. The model did not converge but indicated that there were possible group by session interactions. To simplify the model and focus on the interactions between group and session, a model with only Block 3 was run. That regression model is in Table 4. The fixed effects are shown in Figure 8

The results showed that the Consonant (*coef* = 1.035, *z* = 2.416, *p* < 0.05) and Word Groups (*coef* = 1.657, *z* = 3.603, *p* < 0.001) were on average more accurate during training than the VO Group and that performance was better in later sessions relative to Session 1. This model returned significant negative effects for the Word Group in Sessions 3 (*coef* = −1.113, *z* = −3.922, *p* < 0.001) and 4 (*coef* = −1.227, *z* = −4.064, *p* < 0.001). Figure 8 shows that the Word Group’s improvement in scores between Session 1 and Sessions 3 or 4 was smaller than was the VO Group’s. That is, the Word Group performed near ceiling from the beginning of training, while there was a gradual increase across sessions for the VO Group, which was the reference group. Therefore, the significant negative coefficient is interpreted as a smaller change in score by the Word Group relative to the VO Group.

**Figure 9:**
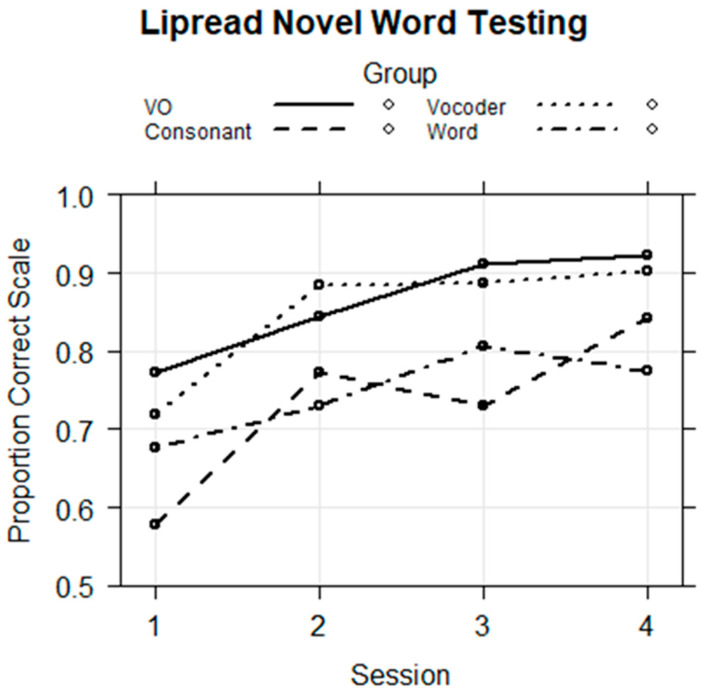
Fixed-Effects Plots: Modeled Novel Word Retention Results for Lipread Novel Word Labeling in Experiment 2.

Novel word lipreading test. Following training, participants were tested on their retention of the labels for nonsense pictures. The results were fitted with a logistic regression model with the fixed factors of group, session, and their interactions and with the random factors of participant and stimulus intercepts. The references were the VO Group and the first session. The regression model is in Table 5. The fixed effects are shown in Figure 9.

The regression model showed that performance was on average higher in Sessions 3 (*coef* = 1.095, *z* = 2.760, *p* < 0.01) and 4 (*coef* = 1.263, *z* = 3.257, *p* < 0.01) than in Session 1. The Consonant Group was on average less accurate than the VO Group (*coef* = −0.905, *z* = −2.724, *p* < 0.01), as Figure 9 shows. Although Figure 9 suggests that the Word Group was also on average less accurate, there was not a significant effect for its mean performance level.

The group by session interactions were of most interest. For Session 2, there was a positive interaction for the Consonant Group (*coef* = 0.424, *z* = 1.992, *p* < 0.05), showing that the group exceed the VO Group’s improvement from Session 1 to Session 2. For Session 3, there was a negative interaction for the Word Group that failed to reach the conventional level *p* < 0.05 of significance, and there was a negative interaction for the Consonant Group that also failed to reach the conventional significance level. However, the Word Group’s accuracy at Session 4 was significantly lower in contrast with the VO Group’s mean difference across sessions (*coef* = −0.761, *z* = −3.150, *p* < 0.01). Figure 9 shows that the VO reference group continued to improve, while the Word Group’s learning curve was shallower.

Pre- and Posttest Forced-choice phoneme identification. Before and after training, participants performed forced-choice phoneme identification with CVCVC stimuli that were not used in training and were not repeated across pre- and posttests. Before analyzing the data for evidence of learning with generalization, we applied the same training performance criteria as in Experiment 1 to screen out participants who may have been inattentive or unable to learn sufficiently for some other reason. If a participant was unable to learn 8 out of 12 words in two or more training sessions by Block 3, their data were not used in the analysis of forced-choice phoneme identification. This eliminated three participants from the Vocoder Group, two from the Word Group, one from the Consonant Group, and four from the VO Group.

The results were fitted with a regression model with the fixed factors of group, test session, consonant position, and all of their higher-way interactions and with the random factors of subject intercepts and the correlated stimulus intercepts and consonant position slopes. The Control Group was the reference group. There were no significant three-way interactions that included consonant position, so the model was reduced in complexity, retaining only the two-way interactions of group by session but retaining all of the random factors.

**Figure 10:**
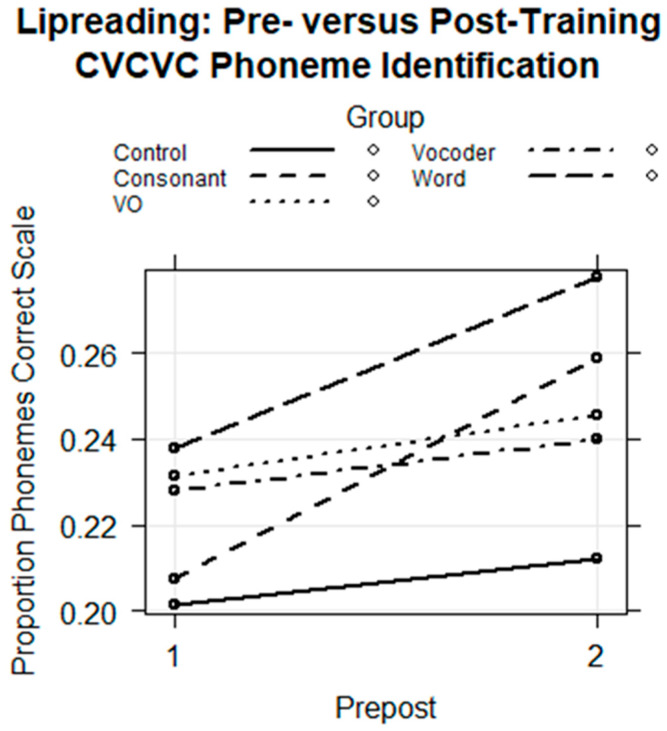
Effects Plot: Modeled Visual Speech Forced-Choice Consonant Identification. The effects are shown for the individual training groups and the Control Group across Pre- and Posttraining.

The final regression model is in Table 6. The fixed effects are shown in Figure 10. There was a significant interaction of Consonant Group with session (*coef* = 0.306, *z* = 2.028, *p* < 0.05). The model was re-run with the fixed-factors position, group, session, and group by session (Table 6). This analysis showed that there was a significant improvement in consonant identification only for the Consonant Group (*coef* = 0.223, *z* = 2.470, *p* < 0.05) relative to the change in pre-to-posttest scores by the Control Group. Figure 10 suggests that the Word Group also improved its phoneme identification scores. However, the comparison with the Control Group shows that the Word Group did not improve enough to attribute its results to training.

**Figure 11:**
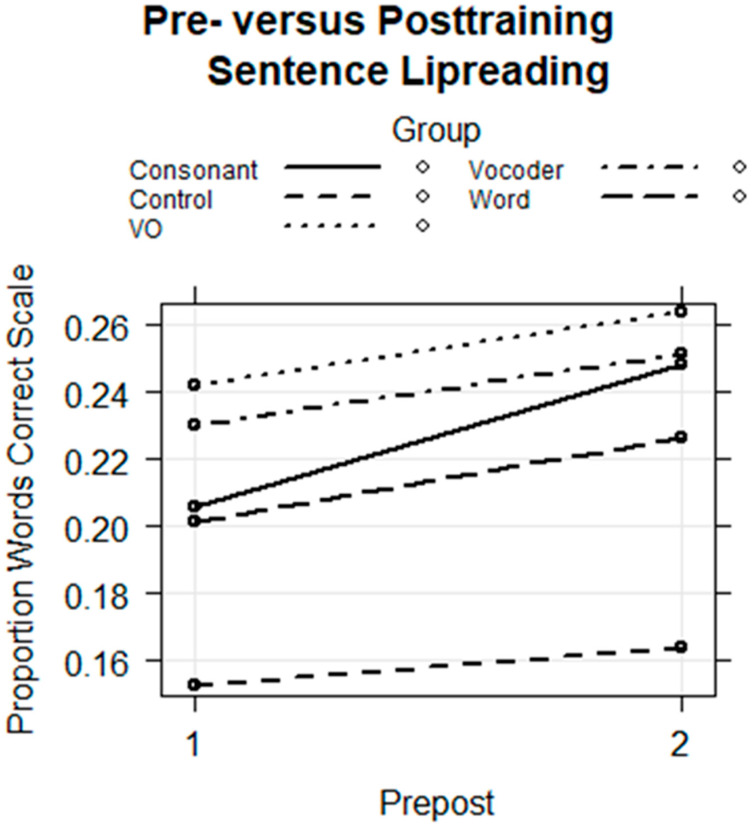
Fixed-Effects Plot: Modeled Sentence Lipreading. The effects are shown for the individual training groups and the Control Group across Pre- and Posttraining sessions.

Pre- and Posttest Sentence lipreading. Before and after training, participants were presented with isolated sentences to lipread and asked to type what they thought the talker had said. The sentences were spoken by a different talker than the one who produced the training stimuli. The results were fitted with a logistic regression model using the proportion of correct words per sentence with the number of stimulus words per sentence as the weights and with a binomial distribution. The model was fitted with the fixed-factors group, session, and group by session, the random effects of subject and stimulus intercepts, and with the Control Group as the reference. The regression model is in Table 6. The fixed effects are shown in Figure 11. The model returned only a coefficient for the interaction of the Consonant Group with session that was not at the conventional statistical significance level (*coef* = 0.153, *z* =1.869, *p* = 0.062) (Table 7). The exponentiated 2.5 to 97.5 confidence bounds for this effect were 0.90 to 1.26, consistent with the elevated *p*-value. Figure 11 shows the quantitatively steeper learning effect for the Consonant Group.

Despite the failure to achieve the conventional *p*-value, we are inclined to interpret this result because the task tested the generalization of learning across talkers, tasks, and stimulus materials and because the results were consistent with our hypotheses. Additionally, the amount of training was small relative to most lipreading training studies [13], and the result encourages additional investigation of this approach.

## 4. Comparison of Novel Word Learning across Experiments

We hypothesized that different types of prior information would affect perceptual learning differently across vocoder versus visual speech training. We were most interested in the word versus consonant contrast because we predicted that word prior information would be effective for promoting vocoder learning, but consonant prior information would be effective for promoting visual speech learning. Experiment 1 showed that the Word Group was more successful in generalization to the phoneme identification task, but the Consonant Group was more successful in Experiment 2. Examination of the novel word retention for vocoded words in Figure 5 and visual spoken words in Figure 9 suggests that there may have been significantly different effects of prior knowledge during training across experiments. In Experiment 1, novel word retention declined across sessions, while in Experiment 2, retention improved. This interaction was tested because it could provide additional insights into the possibility of modality-specific effects during the training task, holding all else constant.

**Figure 12:**
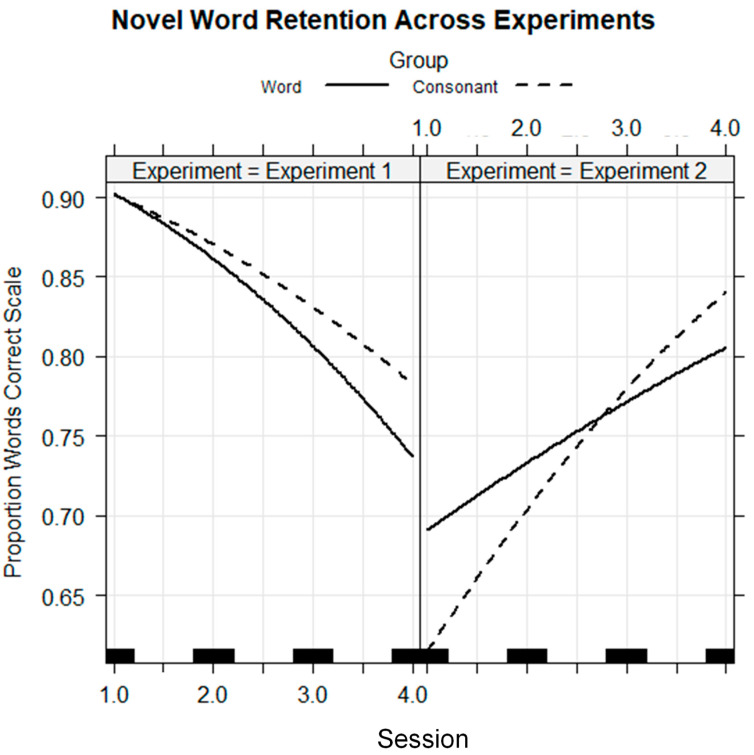
Fixed-Effects Plot: Comparison of Novel Word Retention by Word versus Consonant Groups Across Experiments 1 and 2. Session was coded as a continuous variable to obtain the smooth lines.

To test whether the experience of training with prior word or consonant information had different effects on novel word retention, depending on stimulus modality, we fit a cross-experiment logistic regression model with the fixed-factors group, session, experiment (i.e., Experiment 1, vocoder, vs. Experiment 2, lipreading), and their interactions. Session was coded as a continuous variable to obtain a linear trend. The dependent variable was the individual novel word retention responses (0/1). The participants who were omitted from pre-to-posttest comparisons in their respective experiments were omitted from this analysis also, thus holding training success to be similar across experiments. The random factors were the participant intercepts and the stimulus intercepts and correlated experiment slopes. The random effects took into account that there were different overall levels of performance per participant and that the individual stimuli differed in difficulty between and within experiments. The regression model is in Table 8. The fixed effects are shown in Figure 12.

The model showed that performance decreased on average across sessions (*coef* = −0.396, *z* = −2.974, *p* < 0.01) and that performance in Experiment 2 was on average lower (*coef* = −2.009, *z* = −3.669, *p* < 0.001) (see Figure 12). However, there was an interaction of session with Experiment 2 that was positive (*coef* = 0.602, *z* = 3.606, *p* < 0.001), showing that retention for learning novel lipread words improved significantly in Experiment 2 versus Experiment 1.

Figure 12 shows the downward slope of vocoder retention versus the upward slope of visual speech retention. However, there was no three-way interaction of group by session by experiment. That is, the model returned no differential effect of prior information type as a function of training stimulus modality. However, in Figure 9 for Experiment 2, a crossover can be seen, with higher initial retention and less improvement by the Word Group than by the Consonant Group. In a separate regression model with only the Experiment 2 participants, the interaction of group by session as a continuous variable was significant (*coef* = 0.192, *z* = 2.949, *p* < 0.01). There was no comparable interaction in a separate analysis with the Experiment 1 results. These analyses echo the reporting above (see Table 2 and Table 5) for which session was a categorical variable. Thus, in a direct statistical comparison across training modalities, and with session as a continuous variable, there is evidence for distinctly different performance patterns, holding training task constant.

### Discussion

Experiment 2 followed the design of Experiment 1 with two differences beyond the training modality: in Experiment 2, prior *spoken* words during training were vocoded (Vocoder Group), and the pre- and posttraining tests of generalization included open-set sentence lipreading with a new talker.

During novel word training, the Word Group was highly accurate relative to the VO Group, which improved across sessions. The novel word retention analyses showed that the VO Group retained more novel word labels than did the Word Group. This result was shown with session as a categorical or a continuous variable. This suggests that the prior word information interfered with learning the novel visual spoken words.

The results of the pre- and posttraining consonant identification tests showed that the Consonant Group was unique in significantly improving its scores relative to the Control Group’s test–retest improvement. Additionally, the results of the pre- and posttraining lipreading tests returned evidence that training by the Consonant Group generalized to sentences spoken by a talker who was not seen during training but unfortunately at the level of *p* = 0.062. Although the improvement failed to reach the conventional significance criteria, the result was supportive of our predicted advantage for prior consonant information. Given the brevity of the training and the use of a different talker and task (i.e., lipreading sentences), we think it is important to not ignore this result. Future research is needed to follow up on it.

The results of the VO Group and the Vocoder Group provide additional insights. The VO Group demonstrated that the novel words were visually distinct and can be learned to a high level of accuracy without any prior information, as we have shown previously [56]. The Vocoder Group results suggest that it is not sufficient to reduce information in order to obtain learning but that the remaining information needs to be unambiguous, as was the case with the prior consonant information.

A comparison of novel word retention across experiments, contrasting Word versus Consonant Groups, showed that prior printed information resulted in reduced novel word retention in Experiment 1 and increased retention in Experiment 2. This result strongly supports the suggestion that the performance during a training task may be modality-specific. Likewise, training outcomes demonstrated with the consonant identification tests show that training resulted in different learning as a function of training stimulus modality. Our General Discussion addresses the implications of these findings from the perspectives of RHT versus PCT.

## 5. General Discussion

We hypothesized that different types of prior information during novel word training would have different effects during the novel word retention tests and on generalization following training, depending on stimulus modality. Two training experiments were carried out, one with vocoded speech stimuli (Experiment 1) and one with lipread speech stimuli (Experiment 2). Different groups were assigned to different types of prior information during training: Word Groups saw a printed version of each training stimulus, and Consonant Groups saw only the printed consonants in the words, with underscores replacing the vowels. Additional groups received no prior information (Experiment 1, auditory-only, AO; Experiment 2, visual-only, VO) or a spoken version in a different modality from the training stimuli (Experiment 1, Lipread Group; Experiment 2, Vocoder Group). The spoken prior information was reduced in intelligibility, and so it was a type of control for the consonant-only information. During four separate, approximately 20 min training sessions, participants learned to label 12 novel visual images using 12 novel spoken words. They were tested on their retention of the novel word labeling at the end of each training session. During the retention test, half of the trained words were swapped with new words. Across pre- and posttraining tests, participants carried out forced-choice phoneme identification. In Experiment 2, they also carried out open-set lipreading with a talker who was not seen during training.

In both experiments, the Word Groups had significantly reduced retention of the novel training words relative to the groups that received no prior information. The Word Group in Experiment 1 significantly improved its consonant identification across consonant positions in CVCVC stimuli. The Consonant Group improved on the final consonant position only. However, in Experiment 2, it was the Consonant Group whose training was most effective for improving consonant identification across stimulus positions, and there was evidence that they also improved on open-set lipreading of a talker who was not seen during training. Moreover, in a direct comparison across experiments, the groups that received printed prior information—Word or Consonant—performed differently during the novel word retention: the vocoder trainees became less accurate, and the visual speech trainees became more accurate. Overall, we think the results support our prediction that prior information during training has different effects depending on the modality of the training stimuli.

### 5.1. Modality-Specific Speech Perceptual Learning

Although this was a behavioral study, the predictions that guided its design drew from the neural perceptual learning literature. In the neural literature, the term *feedback* is used to refer to the flow of information through neural hierarchies from higher to lower and is also applied at the cellular level [47]. A crucial point is that external feedback exerts its influence through top-down internal feedback. In behavioral research, the term *feedback* is used to refer to many different methods or displays through which external information is provided to participants *after* they emit a response. The use of prior information here does not qualify for the term “feedback” in the latter conventional sense. However, it does apply to the internal effects of external information.

The technique of presenting prior lexical information during training of vocoded speech has been studied extensively [26,27,35], drawing on the theoretical framework of the PCT for neural systems [34]. The particular PCT explanation of vocoded speech learning we refer to has focused on lexical prediction [17]. Lexical prediction is said to feed back to an internal model of prelexical speech that is compared with bottom-up sensory representations. When the two differ, bottom-up error signals are said to update top-down predictions [26,27,35].

We considered the possibility that prior lexical information could also improve the perception of visual speech. However, visual speech processing differs from vocoded speech beyond its stimulus modality and the different neural pathways that support processing [36,39,40,68]. A speech vocoder reduces and/or distorts the information in the acoustic speech stimulus through a sequence of operations involving first filtering the signal through one or more filters and then recomposing the speech by applying the output amplitude of the filter/s to modulate noise bands or sinusoids that are added together prior to stimulus presentation [20,58]. Perceptual learning appears to be learning of the vocoder transformation, which takes place fairly quickly with prior lexical information and is sufficient to obtain generalization to untrained speech [17]. Importantly, vocoded speech is processed by a highly expert auditory speech processing system.

In contrast, the information that is processed in visual speech is reduced because articulatory mechanisms are only partially visible, and the visual speech processing system of adults with normal hearing is typically inexpert. These factors have supported the widely accepted notion that there are only a small number of distinct visual speech categories, such as one category for /b, p, m/ and another for /ch, dz, sh, zh, d, t/, which are referred to as *visemes* [72,73]. The difficulty in improving lipreading with training would be explained by limitations in the stimulus information, according to the view of visemes as visual speech categories.

However, normal-hearing adults *can discriminate* between phonemes that are commonly grouped within the same viseme [74]. Moreover, in an electrophysiology study, we showed that a visual mismatch negativity response can be elicited with different phonemes from the same viseme [37]. Furthermore, congenitally deaf adults demonstrate access to more prelexical visual speech cues than is typical of adults with normal hearing [28], supporting the existence of phonetic cues that are visible but are not typically learned by normal-hearing adults. Therefore, in contrast with vocoded speech, we suggest that learning to improve visual speech perception requires learning distinctions that are available in the stimulus but not typically processed.

In addition, stimulus selection bias [75] may be in play in maintaining the relatively naïve state of visual speech perceiving in normal-hearing adults. Stimulus selection bias is when stimulus information can be represented and perceived but is not typically processed. *Selection bias* is “an early perceptual bias towards a specific defining feature … such that stimuli with that feature are prioritized over other stimuli during initial encoding. This can be distinguished from the ability to render a stimulus-specific response in the absence of a selection bias” [75] (p. 438). Selection bias can operate to select among information that is too complex and/or too abundant. Selection bias may be operating against everyday learning of visual speech categories because the face conveys socially important information, such as talker identity, sex, mood, and focus of attention. Measures of normal-hearing lipreaders’ eye gaze confirm their extensive attention to the upper portion of the talker’s face [76,77], although eye gaze shifts toward the mouth under noisy conditions [78]. Rewards or salience for attending to social or emotional information may result in stable patterns that select away from attending to visual speech stimuli. There is evidence for a bias toward non-speech social face information such that it captures attention even if it is completely task-irrelevant [79]. Visual speech learning probably involves learning to overcome stimulus selection bias in most adults who have life-long experience of normal hearing, in addition to learning to use additional visual phonetic stimulus information.

Thus, perceptual learning of vocoded versus lipread speech are far different learning problems, in addition to the stimuli being processed through different cortical hierarchies [36,39,40,68]: there is different expertise for vocoded versus visual speech. The vocoded speech is a systematic transformation of overlearned auditory stimuli, and lexical representation is part of expert internal auditory feedforward and feedback pathways. Visual speech is a reduced speech signal that is probably not overlearned and does not engage the expert internal feedforward and feedback pathways exactly the way auditory speech does.

### 5.2. RHT versus PCT

The RHT [47] can be regarded as putting fewer conditions on training than the PCT [17], in that the former requires some conditions that favor an internal reverse search for needed internal stimulus information, while the latter specifies that lexical information is the required condition for enhancing learning. While Davis et al. explain vocoder learning in terms of top-down *lexical* feedback [17], both of our experiments demonstrated learning with only consonant information. The RHT explanation seems consistent with the Consonant Group results in both experiments. Additionally, printed lexical information comprises consonant information, so the PCT explanation needs to show how prior lexical information is different from prior phonemic information.

The results across experiments are, however, consistent with the PCT account in that, independent of training stimulus modality, prior word information reduced novel word learning. This supports the suggestion that prior printed word information effectively engages lexical processing, having a powerful effect on performance. Our study showed that the usefulness of the prior lexical information was specific to vocoder training, which is consistent with the view that lexical knowledge targets auditory speech representations [26,27,35]. However, the current results suggest that additional research is needed to demonstrate the extent to which it is indeed lexical representations and not phonemic information that drive the perceptual learning effect with vocoded speech (c.f., [17]).

### 5.3. Semantic Processing

Results within the RHT framework have been interpreted as evidence that semantic processing interferes with perceptual learning because learning requires relinquishing attention to high-level representations [48]. According to Ahissar (2009), “we cannot have accurate within-category discrimination, with concomitant semantic processing” (p. 288). From the perspective of RHT, if the perceptual learning task requires access to representations that can differentiate within existing perceptual categories (e.g., within ambiguous visual speech categories), then a semantic task will interfere with learning. Research on learning to perceive time-compressed speech in a semantic judgment task supports the view that very little generalizable prelexical learning is achieved with a semantic task [80,81].

We adopted the novel word training approach here because we posited that lipreading training needs to connect prelexical perceptual learning to word recognition while defending against the use of top-down strategies involving existing words. Novel words may defend against top-down lexical feedback to existing auditory representations of real words. Yet our training paradigm involves the semantic task of learning to label novel objects with novel words. The results from the Word Groups suggest that semantic processing does interfere with novel word learning but may not interfere with perceptual learning, depending on the stimulus modality. Additional research is needed to understand how task demands such as semantic processing affect perceptual learning across modalities.

### 5.4. Previous Evidence for Supplying Consonant Information during Lipreading Training with Sentence Stimuli

The current results from the Experiment 2 Consonant Group converge with previous results from a study we carried out on lipreading training with sentence stimuli [14]. That study examined whether the type of external feedback during training significantly affects learning in normal-hearing adults. We considered the implications of the RHT in developing feedback types. We suggested that to obtain learning, (a) there must be stimulus information in bottom-up representations that can be learned, (b) there must be internal feedback to that information, and (c) there must be external feedback that supports learning.

Additionally, we posited that feedback should be contingent on the response, in order for it to be effective [82]. We reasoned that if the whole sentence were printed during training, following an open-set sentence lipreading response, contingency would be low: regardless of the response, the same feedback would be provided. We considered two types of higher-contingency feedback. One type gave feedback for individual correct words in the open-set response and for incorrect words that were nevertheless perceptually near. The trainees could in theory use word feedback for incorrect but perceptually near words to correct prelexical perceptual errors, but the information might also result in reliance on top-down strategies. The other type of feedback was based on the same criteria for correct and near words in the response, but only the consonants were printed for the incorrect but near response words. In that case, contingency would be higher because the trainees could use the consonant feedback to learn their own patterns of consonant errors. Feedback may be more effective when perceivers can learn to predict the valence of the feedback, that is, when they can predict whether they were correct or incorrect [82].

Each type of feedback was assigned to separate training groups, and there was an untrained control group. Before and after training, participants were tested on their forced-choice identification of consonants in CVCVC stimuli (spoken by a different talker than during training), isolated words (spoken by a different talker than during training), and sentences spoken by the same talker (the one used for the sentences in the current study). Words and sentences were presented as VO and in speech-shaped noise, as AO or audiovisual (AV) speech. Training took place in six separate sessions.

The results showed that all of the trainees improved their performance *during* training, but there was no improvement for forced-choice consonant identification, beyond the controls’ improvements across test and retest. The pre-to-posttest scores for isolated words showed a negative effect for the group that received sentence feedback, relative to test–retest improvements on the part of controls, suggesting that sentence feedback may have encouraged top-down strategies such as guessing and relying on lexical knowledge. However, the consonant feedback resulted in significantly increased open-set sentence recognition across all three test modalities, VO, and AO and audiovisual in speech-shaped noise. Mean scores increased significantly by 9.2 percentage points for lipreading, 3.4 percentage points for AO speech in noise, and 9.8 percentage points for audiovisual speech in noise.

The present results thus converged with the earlier ones in showing that learning was fostered by the removal of lexical information and provision of explicit consonant information. However, the results across studies differed in not showing improved forced-choice consonant identification in the earlier study. However, explicit phoneme identification is not a stage in fluent word recognition [83,84]. Lipreading training approaches that train by using phoneme identification typically result in better phoneme identification but provide little benefit to recognizing lipread words [10,11,85]. It is therefore interesting that in the current study, there was evidence for improvements in explicit consonant identification while there was not in the earlier study when the training used sentence stimuli. Furthermore, there was some evidence in the current study for generalization to sentence lipreading.

We recently replicated the sentence training experiment with older adults with hearing loss and again obtained significant evidence that the consonant feedback for incorrect but perceptually near words is more effective than word or sentence feedback [15]. Assuming that successful word recognition entails (implicitly) discriminating phonemic categories [86], it seems that effective visual speech training should somehow induce the trainee to recognize words more accurately by perceptual learning of available but typically unused prelexical visual speech category information.

Thus, we suggest that a general principle of training lipreading may have emerged: effective training requires feedback or prior information that targets prelexical perceptual category information in the context of a task that requires visual speech lexical processing. In other words, there is potentially an important role for visual lexical representations downstream of prelexical visual speech processing. However, top-down auditory lexical representations may interfere with visual speech perceptual learning, although the same information may be highly effective for vocoded speech.

### 5.5. Study Weaknesses

We recognize that further research is needed due to weaknesses in the present study. Pre- and posttests with open-set sentence stimuli were not used to evaluate vocoder learning in Experiment 1 because a task with whole sentences would likely lead to learning that would diminish our ability to observe group effects during the novel word training. Therefore, generalization of learning to sentence stimuli across experiments could not be compared across modalities. However, it would be useful to know whether merely performing open-set sentence identification without feedback would be as effective or significantly different from novel word training with vocoded speech.

In addition, the number of training sessions in this study was low. Moreover, only ~20 min was spent in each training session. The evidence that even this small amount of training with prior consonant information may benefit open-set sentence lipreading for stimuli spoken by a talker who was not seen during training suggests that follow-up research is needed to replicate results and also to determine whether learning continues with additional training sessions. Replication of the open-set sentence lipreading results is needed because the significance of the learning did not achieve the conventional *p* < 0.05 level versus untrained controls but rather was *p* = 0.062.

Finally, we acknowledge the complexity of the study design. Other approaches are needed to obtain converging evidence about the extent to which training and learning differ across modalities.

### 5.6. Conclusions

The patterns of novel word learning by Consonant and Word Groups were different as a function of stimulus modality. The type of information given during training affected perceptual learning as measured across pre- and posttests, depending on the training stimulus modality. Lexical information during novel word training was most effective for vocoded speech, benefitting generalization to phoneme identification. Consonant information was most effective for visual (lipread) speech, benefitting generalization to consonant identification and open-set lipreading. Additionally, performance during training, independent of training stimulus modality, suggests that printed lexical information interferes with being able to learn and retain novel word labeling. This finding demonstrates the automaticity with which printed words are processed and is consistent with the view that top-down feedback automatically targets prelexical auditory representations. Such modality-specific top-down feedback would seem unhelpful in learning to discriminate prelexical information in visual speech, and indeed, the prior consonant information was shown to be more effective for learning visual speech. We conclude that the prior information available to trainees has different effects, depending on the modality of the training stimulus.

## Figures and Tables

**Table 1:** Mixed-effects logistic regression: Novel Word Vocoder Training. Mixed-effects logistic regression with AO Group, Block 1, and Session 1 as references is displayed. Subjects and stimuli are random factors. The coefficients are the log odds.

Effect	Factor	Coefficient	Std. Error	Wald’s z	*p*
Fixed	(Intercept)	−1.227	0.221	−5.558	0.001 ***
	Group Consonant	0.063	0.301	0.210	0.833
	Group Lipread	0.299	0.305	0.978	0.328
	Group Word	0.205	0.310	0.661	0.509
	Session 2	0.435	0.140	3.107	0.002 **
	Session 3	0.827	0.140	5.891	0.000 ***
	Session 4	1.126	0.142	7.913	0.000 ***
	Block 2	1.739	0.109	15.978	0.000 ***
	Block 3	2.791	0.124	22.570	0.000 ***
	Group Consonant × Session 2	0.172	0.138	1.245	0.213
	Group Lipread × Session 2	−0.385	0.142	−2.707	0.007 **
	Group Word × Session 2	0.178	0.143	1.250	0.211
	Group Consonan × Session 3	−0.254	0.160	−1.589	0.112
	Group Lipread × Session 3	−0.614	0.164	−3.744	0.000 ***
	Group Word × Session 3	−0.033	0.165	−0.200	0.841
	Group Consonan × Session 4	−0.282	0.165	−1.716	0.086 ^•^
	Group Lipread × Session 4	−0.820	0.168	−4.891	0.000 ***
	Group Word × Session 4	−0.455	0.169	−2.685	0.007 **
	Group Consonant × Block 2	−0.168	0.119	−1.410	0.159
	Group Lipread × Block 2	−0.036	0.121	−0.300	0.764
	Group Word × Block 2	−0.233	0.123	−1.892	0.059 ^•^
	Group Consonant × Block 3	−0.193	0.137	−1.416	0.157
	Group Lipread × Block 3	0.327	0.146	2.244	0.025 *
	Group Word × Block 3	−0.243	0.142	−1.718	0.086 ^•^
	Session 2 × Block 2	0.215	0.116	1.853	0.064 ^•^
	Session 3 × Block 2	0.268	0.117	2.278	0.023 *
	Session 4 × Block 2	0.252	0.119	2.122	0.034 *
	Session 2 × Block 3	−0.037	0.131	−0.284	0.776
	Session 3 × Block 3	−0.154	0.132	−1.173	0.241
	Session 4 × Block 3	0.184	0.141	1.306	0.191
Random	Factor	Variance	Standard Deviation		
	Subject Intercept	0.669	0.818		
	Stimulus Intercept	0.036	0.191		

Note: ***, *p* < 0.001; **, *p* < 0.01; *, *p* < 0.05; and ^•^ *p* < 0.10.

**Table 2:** Mixed-effects logistic regression: Novel Word Test. Mixed-effects logistic regression with AO Group and Session 1 as references. Subjects and stimuli are random factors. The coefficients are the log odds.

Effect	Factor	Coefficient	Std. Error	Walds’ z	*p*
Fixed	(Intercept)	1.836	0.389	4.726	0.000 ***
	Group Consonant	−0.322	0.433	−0.743	0.457
	Group Lipread	0.463	0.449	1.030	0.303
	Group Word	−0.186	0.447	−0.416	0.677
	Session 2	0.189	0.398	0.476	0.634
	Session 3	0.766	0.409	1.875	0.061 ^•^
	Session 4	0.786	0.411	1.912	0.056 ^•^
	Group Consonant × Session 2	0.263	0.295	0.890	0.373
	Group Lipread × Session 2	−0.303	0.315	−0.962	0.336
	Group Word × Session 2	−0.012	0.299	−0.041	0.968
	Group Consonant × Session 3	−0.997	0.447	−2.229	0.026 *
	Group Lipread × Session 3	−0.966	0.466	−2.073	0.038 *
	Group Word × Session 3	−1.172	0.451	−2.597	0.009 **
	Group Consonant × Session 4	−1.085	0.448	−2.420	0.016 *
	Group Lipread × Session 4	−1.465	0.462	−3.169	0.002 **
	Group Word × Session 4	−1.526	0.453	−3.369	0.001 ***
Random	Factor	Variance	Standard Deviation		
	Subject Intercept	1.243	1.115		
	Stimulus Intercept	0.326	0.571		

Note: ***, *p* < 0.001; **, *p* < 0.01; *, *p* < 0.05; and ^•^ *p* < 0.10.

**Table 3:** Pre- and Posttraining Forced-Choice Consonant Identification with Vocoded CVCVC Stimuli. Mixed-effects logistic regression with the Control Group, consonant Position 1, and Pretraining sessions as references. Subjects and stimuli are random intercepts, and consonant positions are correlated random slopes. The coefficients are the log odds.

Effect	Factor	Coefficient	Std. Error	Walds’ z	*p*
	(Intercept)	−1.269	0.266	−4.769	0.000 ***
	Prepost Post	0.492	0.169	2.919	0.004 **
	Group AO	−0.163	0.234	−0.697	0.486
	Group Consonant	−0.916	0.249	−3.678	0.000 ***
	Group Lipread	−1.053	0.255	−4.135	0.000 ***
	Group Word	−0.848	0.261	−3.255	0.001 **
	Consonant Position 2	0.962	0.266	3.621	0.000 ***
	Consonant Position 3	0.392	0.277	1.412	0.158
	Prepost Post × Group AO	0.232	0.216	1.077	0.282
	Prepost Post × Group Consonant	0.098	0.229	0.428	0.669
	Prepost Post × Group Lipread	−0.005	0.237	−0.022	0.983
	Prepost Post × Group Word	0.492	0.237	2.072	0.038 *
	Prepost Post × Position 2	0.024	0.223	0.107	0.915
	Prepost Post × Position 3	−0.138	0.226	−0.610	0.542
	Group AO × Position 2	−0.004	0.205	−0.019	0.985
	Group Consonant × Position 2	0.388	0.228	1.703	0.089 ^•^
	Group Lipread × Position 2	0.315	0.235	1.340	0.180
	Group Word × Position 2	0.736	0.236	3.120	0.002 **
	Group AO × Position 3	0.019	0.209	0.091	0.927
	Group Consonant × Position 3	0.025	0.233	0.106	0.916
	Group Lipread × Position 3	0.036	0.241	0.151	0.880
	Group Word × Position 3	0.918	0.237	3.882	0.000 ***
	Prepost Post × Group AO × Position 2	−0.077	0.285	−0.268	0.788
	Prepost Post × Group Consonant × Position 2	0.072	0.300	0.239	0.811
	Prepost Post × Group Lipread × Position 2	0.114	0.309	0.368	0.713
	Prepost Post × Group Word × Position 2	−0.057	0.310	−0.184	0.854
	Prepost Post × Group AO × Position 3	0.069	0.289	0.238	0.812
	Prepost Post × Group Consonant × Position 3	0.674	0.304	2.213	0.027 *
	Prepost Post × Group Lipread × Position 3	0.325	0.315	1.033	0.302
	Prepost Post × Group Word × Position 3	−0.014	0.311	−0.044	0.965
Random	Factor	Variance	Standard Deviation		
	Stimulus Intercept	3.537	1.881		
	Position 2 Slope	4.286	2.070		
	Position 3 Slope	4.800	2.191		
	Subject Intercept	0.187	0.432		

Note: ***, *p* < 0.001; **, *p* < 0.01; *, *p* < 0.05; and ^•^ *p* < 0.10.

**Table 4:** Novel Word Lipreading Training. Mixed-effects logistic regression with VO Group and Session 1 as references. Subjects and stimuli are random factors. The coefficients are the log odds.

Effect	Factor	Coefficient	Std. Error	Walds’ z	*p*
Fixed	(Intercept)	1.176	0.315	3.730	0.000 ***
	Group Consonant	1.035	0.428	2.416	0.016 *
	Group Vocoder	0.231	0.444	0.522	0.602
	Group Word	1.657	0.460	3.603	0.000 ***
	Session 2	0.500	0.178	2.802	0.005 **
	Session 3	1.012	0.192	5.282	0.000 ***
	Session 4	1.428	0.208	6.878	0.000 ***
	Group Consonant × Session 2	0.445	0.262	1.698	0.089 ^•^
	Group Vocoder × Session 2	0.303	0.244	1.242	0.214
	Group Word × Session 2	−0.030	0.291	−0.103	0.918
	Group Consonant × Session3	0.236	0.283	0.836	0.403
	Group Vocoder × Session 3	0.105	0.261	0.401	0.688
	Group Word × Session 3	−1.113	0.284	−3.924	0.000 ***
	Group Consonant × Session 4	−0.074	0.298	−0.247	0.805
	Group Vocoder × Session 4	0.038	0.283	0.136	0.892
	Group Word × Session 4	−1.227	0.302	−4.064	0.000 ***
	Factor	Variance	Standard Deviation		
Random	Subjects Intercepts	1.681	1.297		
	Stimulusi Intercepts	0.029	0.171		

Note: ***, *p* < 0.001; **, *p* < 0.01; *, *p* < 0.05; and ^•^ *p* < 0.10.

**Table 5:** Lipreading Novel Word Test. Mixed-effects logistic regression with VO Group and Session 1 as references. Subjects and stimuli are random factors. The coefficients are the log odds.

Effect	Factor	Coefficient	Std. Error	Walds’ z	*p*
Fixed	(Intercept)	1.224	0.353	3.470	0.001 **
	Group Consonant	−0.905	0.332	−2.724	0.006 **
	Group Vocoder	−0.281	0.356	−0.791	0.429
	Group Word	−0.489	0.347	−1.408	0.159
	Session 2	0.474	0.389	1.218	0.223
	Session 3	1.095	0.397	2.760	0.006 **
	Session 4	1.263	0.400	3.157	0.002 **
	Group Consonant × Session 2	0.424	0.213	1.992	0.046 *
	Group Vocoder × Session 2	0.620	0.244	2.544	0.011 *
	Group Word × Session 2	−0.213	0.221	−0.966	0.334
	Group Consonant × Session 3	−0.421	0.226	−1.865	0.062 ^•^
	Group Vocoder × Session 3	0.028	0.256	0.108	0.914
	Group Word × Session 3	−0.408	0.238	−1.714	0.087 ^•^
	Group Consonant × Session 4	0.092	0.237	0.387	0.699
	Group Vocoder × Session 4	0.019	0.264	0.073	0.942
	Group Word × Session 4	−0.761	0.242	−3.150	0.002 **
Random	Factor	Variance	Standard Deviation		
	Subjects Intercepts	0.995	0.998		
	Stimulus Intercepts	0.371	0.609		

Note: **, *p* < 0.01; *, *p* < 0.05; and ^•^ *p* < 0.10.

**Table 6:** Pre- and Posttraining Forced-Choice Consonant Identification with Lipread CVCVC Stimuli. Mixed-effects logistic regression with VO Group and pretest session as references. Subjects and stimuli are random factors. The coefficients are the log odds.

Effect	Factor	Coefficient	Std. Error	Walds’ z	*p*
	(Intercept)	−1.095	0.213	−5.146	0.000 ***
	Prepost Post	0.066	0.069	0.960	0.337
	Group Consonant	0.037	0.133	0.281	0.779
	Group VO	0.178	0.144	1.232	0.218
	Group Vocoder	0.159	0.144	1.099	0.272
	Group Word	0.213	0.140	1.522	0.128
	Position 2	−0.223	0.206	−1.082	0.279
	Position 3	−0.628	0.229	−2.739	0.006 **
	Prepost Post × Group Consonant	0.223	0.090	2.470	0.013 *
	Prepost Post × Group VO	0.013	0.098	0.135	0.893
	Prepost Post × Group Vocoder	0.000	0.098	−0.002	0.999
	Prepost Post × Group Word	0.142	0.094	1.502	0.133
Random	Factor	Variance	Standard Deviation		
	Subjects Intercepts	0.143	0.378		
	Stimulus Intercepts	1.666	1.291		
	Stimulus Position 2 Slopes	1.965	1.402		
	Stimulus Position 3 Slopes	2.444	1.563		

Note: ***, *p* < 0.001; **, *p* < 0.01; *, *p* < 0.05.

**Table 7:** Pre- and Posttraining Open-Set Lipreading. The coefficients are the log odds.

Effect	Factor	Coefficient	Std. Error	Walds’ z	*p*
Fixed	(Intercept)	−1.716	0.285	−6.023	0.000 ***
	Group Consonant	0.367	0.291	1.264	0.206
	Group VO	0.574	0.316	1.820	0.069 ^•^
	Group Vocoder	0.508	0.316	1.609	0.108
	Group Word	0.338	0.307	1.100	0.271
	Prepost 2	0.089	0.063	1.405	0.160
	Group Consonant × Prepost 2	0.153	0.082	1.869	0.062 ^•^
	GroupVO × Prepost 2	0.026	0.087	0.302	0.763
	GroupVocoder × Prepost 2	0.029	0.088	0.329	0.742
	GroupWord × Prepost 2	0.063	0.087	0.723	0.469
Random	Factor	Variance	Standard Deviation		
	Subjects Intercepts	0.856	0.925		
	Stimulus Intercepts	2.366	1.538		

Note: ***, *p* < 0.001; ^•^ *p* < 0.10.

**Table 8:** Novel Word Retention by Word versus Consonant Groups Across Experiments 1 and 2. The coefficients are the log odds.

Effect	Factor	Coefficient	Std. Error	Wald’s z	*p*
Fixed	(Intercept)	2.610	0.439	5.952	0.000 ***
	Group Consonant	−0.093	0.418	−0.224	0.823
	Session	−0.396	0.133	−2.974	0.003 **
	Experiment 2	−2.009	0.548	−3.669	0.000 ***
	Group Consonant × Session	0.085	0.093	0.915	0.360
	Group Consonant × Experiment 2	−0.437	0.514	−0.851	0.395
	Session × Experiment 2	0.602	0.167	3.606	0.000 ***
	Group Consonant × Session × Experiment 2	0.107	0.114	0.945	0.345
Random		Variance	Standard Deviation	Correlation	
	Subjects Intercepts	0.686	0.828		
	Stimulus Intercepts	0.387	0.622		
	Stimulus Experiment Slopes	0.621	0.788	−0.49	

Note: ***, *p* < 0.001; **, *p* < 0.01.

## Data Availability

The data presented in this study are openly available in the Open Science Framework at https://osf.io/v5z8x/ accessed 25 June 2023. The datasets generated during and/or analyzed during the current study are available along with the R code on the Open Science Framework platform.

## Appendix A

Lexical and consonant prior information used in training and transcriptions of the training and test words. The transcriptions, which are shown here in ARPABET [60], a single-character phonemic transcription system in ASCII, were used in computationally generating the stimuli. For example, /sICUd/ was presented as “s_ch_d” during training with consonant information, and “sitchud” was used during training with lexical prompts. The transcriptions listed under Test include the six words retained from training in the first part of the list and the six new words in the second part of the list.

**Table A1:** Lexical and consonant prior information used in training and transcriptions of the training and test words.

List							
1				2			
Training			Test	Training			Test
Transcription	Lexical	Consonant	Transcription	Transcription	Lexical	Consonant	Transcription
sICUd	sitchud	s_ch_d	sICUd	mITak	mithock	m_th_k	mITak
pcriD	pawreethe	p_r_th	pcriD	lRman	lerman	l_rm_n	lRman
CRfIG	cherfing	ch_rf_g	CRfIG	Sczxn	shawzun	sh_z_n	Sczxn
wInct	winnaught	w_n_t	wInct	bodut	bodute	b_d_t	bodut
kUmxl	cummle	k_m_l	kUmxl	ridap	reedop	r_d_p	ridap
hUbIG	hoobing	h_b_g	hUbIG	zEriC	zereach	z_r_ch	zEriC
digaz	deegoz	d_g_z	SEsxl	pIDRz	pitherz	p_th_rz	pEt^f
lIZxs	lizzhus	l_zh_s	bozEn	wRsIG	wersing	w_rs_g	f@Jxs
mcTxs	mothis	m_th_s	JovRs	k@fRt	kaffert	k_f_rt	viw^s
tETan	tethon	t_th_n	m@tuT	TEmat	themat	th_m_t	nIsxJ
rip^J	repudge	r_p_j	fctab	dib^J	debudge	d_b_j	JUkiz
yulat	yulot	y_l_t	D@zxk	sEJud	sejude	s_j_d	wEsxJ
List							
3				4			
Training			Test	Training			Test
Transcription	Lexical	Consonant	Transcription	Transcrip-tion	Lexical	Consonant	Transcription
hIluz	hiluze	h_l_z	hIluz	kizxl	keezul	k_z_l	kizxl
Cudxk	choodik	ch_d_k	Cudxk	wEsIk	wesick	s_s_k	wEsIk
bUran	burran	b_r_n	bUran	bincl	beenawl	b_n_l	bincl
Jobxt	jobit	j_b_t	Jobxt	pcgxs	pauguss	p_g_s	pcgxs
m@fis	mafeese	m_f_z	m@fis	TuSxz	thewshes	th_sh_s	TuSxz
kcraC	coratch	c_r_tch	kcraC	s@bad	sabbod	s_b_d	s@bad
tEfRk	tefferk	t_f_rk	zEnop	yupan	yupon	y_p_n	m@d^v
ncrim	naureem	n_r_m	dik^p	hob^k	hoabuk	h_b_k	SRfxn
ril^n	reelun	r_l_n	yUS^k	dISxp	dishup	d_sh_p	l@kat
TIfxs	thifuss	th_f_s	rIZxl	vIpxd	vipid	v_p_d	zESxm
fICUt	fitchut	f_ch_t	lctak	m@Jxv	madjuv	m_j_v	CIlxz
S@dxz	shaddiz	sh_d_z	w@vxt	nupis	nupeese	n_p_z	fEkRz
